# Benchmarking LiDAR and LiDAR-Inertial Odometry Algorithms for Embedded UAV Deployment in Warehouse Environments

**DOI:** 10.3390/s26206568

**Published:** 2026-10-16

**Authors:** António T. Matos, André Dias, André Moura, João J. Martins, Ricardo Morais, José Almeida

**Affiliations:** CRAS—Center for Robotics and Autonomous Systems, INESC TEC—Institute for Systems and Computer Engineering, Technology and Science, Campus da FEUP, Rua Dr. Roberto Frias, 4200-465 Porto, Portugal; andre.dias@inesctec.pt (A.D.); andre.f.moura@inesctec.pt (A.M.); joao.p.pires@inesctec.pt (J.J.M.); ricardo.morais@inesctec.pt (R.M.); jose.m.almeida@inesctec.pt (J.A.)

**Keywords:** LiDAR-inertial odometry, SLAM, 3D LiDAR, unmanned aerial vehicles, GNSS-denied navigation, embedded computing, computational latency, sensor degeneracy, indoor logistics

## Abstract

**Highlights:**

**What are the main findings?**

- Evaluation of open-source LiDAR odometry and LiDAR-inertial odometry systems on flight data from a laboratory and an operational warehouse, on a workstation and on the vehicle’s own embedded computer: on the embedded computer, laboratory trajectory error changed by at most 3 mm for every system that consumed its full input, whereas time per scan grew 1.8 to 3.9 times and the cost ranking reordered.
- At the laboratory, the only site with a metric reference, the selected LiDAR-inertial system reduced the typical trajectory error to about a third of that of the onboard visual-inertial odometry.

**What are the implications of the main findings?**

- One system discarded up to two-thirds of its input while latency, coverage and memory continued to report normal operation. A benchmark that reports a rate should also report the fraction of input that produced it.
- A cost ranking obtained on a workstation does not survive the move to the target, so the systems have to be chosen on the hardware they will run on.

**Abstract:**

Reliable LiDAR-inertial odometry is central to UAV navigation in GNSS-denied indoor logistics, yet most benchmarks target ground vehicles and ignore the compute limits of aerial platforms. We benchmark six systems spanning two modalities and three estimator families—LiDAR-only (F-LOAM, KISS-ICP) and, within tightly coupled LiDAR-inertial estimation, filter-based (FAST-LIO2, Faster-LIO), graph-optimisation-based (liorf, from LIO-SAM) and geometric-observer-based (DLIO)—on flight data from a motion-capture laboratory and an operational warehouse. We identify and correct four ways a per-scan latency figure misleads; one misstates a system’s cost fourfold. When identical data are replayed on a workstation and on the vehicle’s computer, accuracy transfers to within 3 mm, while cost does not: the penalty tracks how far an implementation distributes its work, and on the passively cooled target it moves by up to a factor of two between sessions whose trajectories agree within millimetres. The workstation’s most accurate system collapses on the target, silently discarding up to two-thirds of its input while latency, coverage and memory report normal operation. Of the two systems that stay inexpensive, within budget and consistent with the reference, Faster-LIO is the cheaper; used as the vehicle’s position source, it reduced the onboard visual-inertial estimator’s typical error on all eight closed-loop flights.

## 1. Introduction

Warehouse and intralogistics operations are increasingly adopting automation technologies to enhance operational efficiency, reduce costs, and improve safety [1]. Autonomous Unmanned Aerial Vehicles (UAVs) are an attractive solution for various applications, such as inventory management, monitoring, and inspection tasks [2,3]. However, deploying UAVs in indoor logistics environments presents a challenge in localisation: Global Navigation Satellite System (GNSS) signals are often unavailable or unreliable due to signal occlusion and multipath effects, requiring the vehicle pose to be estimated entirely from onboard sensors.

Light Detection and Ranging (LiDAR) sensing is particularly well-suited for these environments, as it measures range directly, is invariant to illumination, and captures the aisle and rack geometry that constrains a drifting estimate [4]. Pairing it with an Inertial Measurement Unit (IMU), which bridges the interval between scans and provides per-point motion compensation, yields a LiDAR-Inertial Odometry (LIO) system. This work addresses the use of LiDAR Odometry (LO) and LIO systems for indoor UAV navigation on embedded hardware.

Demonstrated UAV deployments in warehouses and industrial plants bound drift with pre-installed fiducial markers or with a map built during an earlier manual flight [2,3,5], leaving open whether unaided LiDAR odometry is sufficient. The data on which LiDAR odometry is evaluated is generally carried by cars, handheld rigs and wheeled robots through street, campus, construction and laboratory settings [6,7,8,9], whereas the benchmark data recorded in flight carries no LiDAR at all [10]. None of these scenarios exercise the condition that defines this deployment, namely shelf rows that lack distinctive geometric features and repeat along the direction of travel [2]; accuracy attained on such data is therefore not evidence of accuracy attainable here, and the head-to-head comparisons built on it inherit the same limitation [9,11,12,13,14,15].

Those comparisons evaluate the accuracy of the pose estimate against a reference, but the computational cost of the system, meaning the time taken to process a scan and the processor and memory occupied while doing it, is frequently absent altogether, and, in the studies that do report it, takes the form of processor and memory occupancy [11,12]. Some benchmarks also exclude these metrics deliberately, on the grounds that they depend on the hardware platform and runtime environment [9]. The methodology for judging accuracy and cost jointly does exist, and treats speed and even energy as first-class metrics alongside error [16,17]; it was, however, developed for visual pipelines, and its embedded validation has been carried out on boards equipped with Graphics Processing Units (GPUs) [16,18]. System selection for a compute-constrained aerial platform therefore rests on desktop rankings whose transfer to the target is assumed rather than measured.

This work addresses that gap by evaluating six open-source LO and LIO systems under a single protocol. Two are the LiDAR-only baselines F-LOAM [19] and KISS-ICP [20], and four are tightly coupled systems spanning the three estimator families reviewed in Section 2.1.2, namely FAST-LIO2 [21], Faster-LIO [22], LIO-SAM [23] in the liorf [24] formulation, and DLIO [25]. Flight recordings from an operational warehouse and from a laboratory with motion capture were replayed at the sensor rate, first on a workstation and then on the vehicle’s embedded computer. Faster-LIO was then flown in closed loop, in place of the onboard visual-inertial estimator, on eight laboratory flights over four trajectories. The main contributions are as follows:

- **An evaluation on flight data from a laboratory and an operational warehouse.** To the best of our knowledge, no public LiDAR odometry dataset with ground truth has been recorded in a racked warehouse, from any carrier, so the sequences used here were collected for this study. Accuracy is quantified at the laboratory site, which is instrumented with motion capture; the warehouse sequences carry no metric reference, are held out from tuning, and serve to test whether the conclusions survive rather than to rank accuracy.
- **A latency measurement procedure that is comparable across architectures.** We identify four ways in which a per-scan latency figure misleads—structural input buffering read as computation, unbounded queue growth that swamps the work it is meant to measure, throughput scored against a package’s own output rather than against the sensor period, and truncated runs that every self-reported metric scores as complete—and correct each in the measurement tooling. The first alone misstates one package’s cost by a factor of four and reverses its rank against another.
- **Validation on the deployment target.** Accuracy, processor and memory use, and per-scan latency are reported on the flight computer itself rather than on a desktop proxy. We are not aware of any peer-reviewed characterisation of this platform under sustained robotics load.
- **A characterisation of behaviour under a hard compute ceiling**, including a failure mode in which a system discards a large fraction of its input while conventional health indicators continue to report normal operation. It appears on every warehouse sequence, and at the laboratory in the warmer of the two sessions.
- **A comparison with the onboard Visual-Inertial Odometry (VIO). ** At the laboratory, Faster-LIO is scored against the motion-capture reference alongside the vehicle’s visual-inertial estimator, first replayed on the drone computer and then flown in closed loop in place of that estimator.

The results show that accuracy transfers from the workstation to the flight computer for every system fast enough to consume its input, whereas computational cost does not, and the penalty is largest for the implementations that distribute their work across threads. Under the compute ceiling of the onboard computer, KISS-ICP consumes between a third and a half of the incoming scans on every warehouse recording while latency, coverage, and memory continue to report nominal operation, so that only the fraction of input consumed reveals the failure. Faster-LIO stays within the sensor budget at both sites on both machines, and at the laboratory its typical trajectory error is about a third of that of the onboard VIO.

The remainder of the paper is organised as follows. Section 2 reviews related work; Section 3 describes the platform, the sites, the systems under test, the tuning protocol and the measurement methodology; Section 4 presents the results; Section 5 discusses their implications for deployment and the limitations of the study; and Section 6 concludes.

## 2. Related Work

### 2.1. LiDAR Odometry Systems

**Figure 1:**
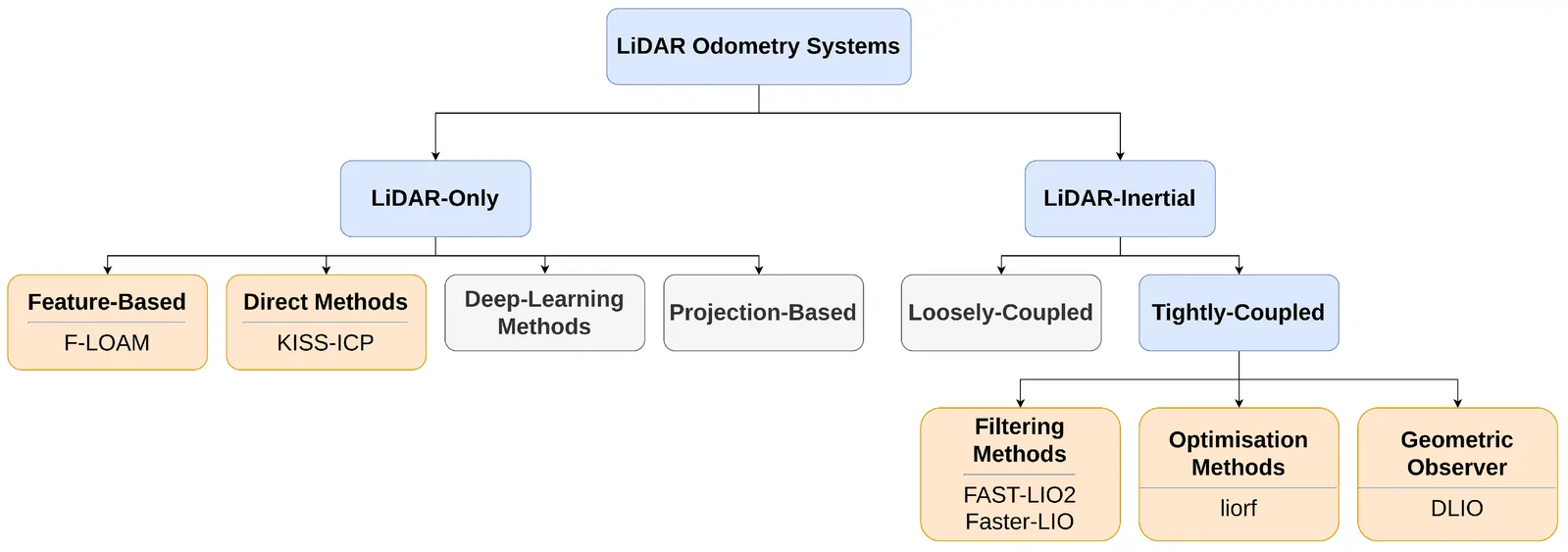
Taxonomy of LiDAR odometry systems and the six evaluated systems in this work. Orange nodes are the families evaluated here, each with the systems that represent it, grey nodes are recognised families that fall outside the scope of this study, and blue nodes are the parent categories under which they are grouped. Synthesised from [4,26].

LiDAR odometry systems can be distinguished by the sensor modality they use and, within each modality, by the mechanism that produces the estimate [4,26]. Figure 1 places the six systems evaluated in this work within that taxonomy. Following the terminology of Cadena et al. [27], we distinguish odometry, incremental motion estimation with no global correction, from Simultaneous Localisation and Mapping (SLAM), in which loop closure enforces a globally consistent map. Under this criterion, five of the six evaluated systems are odometry pipelines, and only one performs SLAM.

#### 2.1.1. LiDAR-Only Methods

LiDAR-only odometry systems estimate a robot’s motion from consecutive LiDAR scans alone [4]. They are classified by how those scans are matched, as Figure 1 shows; of the families it lists, two are represented among the systems evaluated here, feature-based matching and direct matching.

Feature-based matching approaches [28] extract feature points from the LiDAR point clouds and estimate the relative motion transformation from those points alone. Fewer points enter the scan matching, which is reported to improve computational speed, and the points that do are selected for their physical significance. This method may, however, be ineffective in featureless and dynamic environments [4,26]. Among the systems evaluated in this work, F-LOAM [19] adopts this formulation. Its authors further propose a non-iterative two-stage distortion compensation and an adaptive weighting of the extracted features, neither of which the evaluated implementation performs (Section 3.4).

Direct matching methods estimate the robot’s motion from the point cloud itself, without extracting features. This makes them well-suited to featureless environments, where the primitives feature-based methods depend on are scarce, while also preserving detail that feature extraction discards. The cost is that the number of points entering the correspondence search is no longer reduced, leading to an increase in computation time. The registration is typically performed by Iterative Closest Point (ICP) [29] or a variant of it. Among the systems evaluated in this work, KISS-ICP [20] adopts this formulation and is presented by its authors as requiring no per-dataset tuning.

#### 2.1.2. LiDAR-Inertial Methods

LiDAR-Inertial Odometry systems emerged to address LiDAR-related issues, such as motion distortion [4]. The IMU provides angular velocity and linear acceleration at a rate far higher than the LiDAR scan rate, which allows the motion during a scan to be compensated and constrains the estimate between scans. The two sources may be fused loosely, where each sensor produces an estimate that is combined afterwards, or tightly, where both sets of measurements enter a single estimation problem. Tightly coupled methods achieve higher accuracy and robustness in complex scenes [26,30], at a higher computational cost, and form the only branch represented among the systems evaluated here.

Within the tightly coupled branch, filter-based methods emerged in response to the growing computational demands of graph-based approaches, offering a lighter alternative based on Kalman filtering [4]. They construct state and measurement models from the sensor observations and achieve good real-time performance while using every LiDAR scan [26]. The corresponding limitation is that they do not incorporate loop closure constraints, so error accumulation becomes considerable in large-scale scenes. Two of the systems evaluated in this work adopt this formulation, FAST-LIO2 [21] and Faster-LIO [22].

Graph optimisation methods express the sensor observations as factors in a factor graph and solve the resulting Maximum a Posteriori (MAP) problem [26]. This formulation suppresses accumulated error more effectively than filtering, and it admits factors that a filter cannot represent, such as loop closure [26]. The cost is computational, and to operate in real time, these methods restrict the optimisation to keyframes, so that only a subset of the scans enters the graph [26]. LIO-SAM [23] adopts this formulation, whilst the system evaluated in this work is its liorf fork [24], described in Section 3.

A third approach replaces the filter and the graph with a geometric observer, a nonlinear estimator designed so that its convergence can be established analytically [25]. Its authors present the resulting convergence guarantee as the main advantage over filtering and pose graph optimisation [25,31]. The observer nonetheless treats the pose returned by scan matching as a measurement, so the front end remains the limiting factor for accuracy. Although the literature commonly divides tightly coupled methods into filter-based and optimisation-based formulations only [26], the geometric observer is treated here as a separate family, since DLIO [25], one of the systems evaluated in this work, is built on it.

#### 2.1.3. Systems Evaluated in This Work

The six systems evaluated in this work were selected under three sets of criteria. The first is imposed by the target platform described in Section 3, so each system must be publicly available as source code, must build and run under Robot Operating System (ROS) Noetic on an aarch64 Central Processing Unit (CPU) and, where it uses inertial data, must operate with the 6-axis IMU embedded in the LiDAR. The second is imposed by the taxonomy of Figure 1, which requires at least one representative of each family considered, so that the comparison separates architectures instead of implementations of a single architecture. The third concerns relevance, as all six systems are treated in recent surveys of the field [4,26], and each appears in at least one recent independent comparative evaluation [9,13,14], which establishes that its implementation is one that others obtain, build and run. The comparison is therefore drawn over systems a practitioner would plausibly deploy rather than over an arbitrary sample. One family is represented by two systems by design, as FAST-LIO2 [21] and Faster-LIO [22] share the same estimator but differ in the structure used to store and search the map, isolating the effect of that structure from every other design choice. The graph optimisation family is represented by liorf instead of LIO-SAM itself because the original implementation requires a 9-axis IMU [32].

Table 1 summarises the design of the six systems along the axes used in the comparison. Three of the four LIO systems form point-to-plane correspondences, so liorf and FAST-LIO2 share the residual family and differ in the estimator that consumes it and in how the map is maintained. Only liorf is capable of loop closure, and therefore of correcting accumulated error.

### 2.2. Aerial Deployment in Indoor Logistics

Autonomous flight in warehouses and comparable industrial facilities has been demonstrated, and in each case the pose estimate is anchored by something external to the vehicle’s own motion.

Beul et al. [2] fly a commercial platform carrying a 3D LiDAR through the aisles of an operating warehouse, but the pose graph is initialised with a map recorded on an earlier manual flight, so the estimate is constrained by a map the vehicle did not acquire on this one. The AprilTags and RFID tags on the stock serve the inventory task and do not enter the pose estimate.

Kwon et al. [3] fuse a 2D LiDAR, a 1D range sensor for altitude, an IMU and three cameras in an Extended Kalman Filter (EKF), with the absolute correction coming from AprilTags read against a map of known tag poses. The tags spare the vehicle a mapping flight, but replace the prior map rather than remove the need for one, and two of the cameras, facing the ceiling and the floor, are there to counter drift that the aisle itself induces. The authors report a localisation error of 3.12 cm on average and 25.68 cm at maximum, in aisles 1.8 m wide and up to 60 m long.

Perez-Grau et al. [5] address a large manufacturing facility instead of a warehouse and arrive at the same arrangement, with a 2D LiDAR providing odometry by ICP and a separate global alignment against a static map, computed on earlier flights, correcting the accumulated drift.

Two further systems avoid the prior map, but not the anchor. Martinez-Carranza and Rojas-Perez [33] localise a micro air vehicle with a monocular camera and an altimeter and no markers in the environment, synthesising a depth image from the altitude readings so that an RGB-D formulation of ORB-SLAM can recover metric scale. The estimate holds while the floor is planar, an assumption the authors deem reasonable for warehouses. Dias et al. [34], whose platform is the one used in this work, also fly without a prior map, using visual-inertial odometry, and rely on fiducial markers whose detection drives a relocalisation layer that bounds the drift over a rack-scanning flight.

Across these systems, the anchor takes one of three forms: a map acquired before the mission, fiducial markers installed in the building, or an assumption about the building itself. None of the five vehicles are left to localise from their own motion alone. To the best of our knowledge, neither accuracy nor computational cost of unaided 3D LiDAR odometry in a racked warehouse has been reported [2,3,5,33,34], which is the question addressed here.

### 2.3. Comparative Evaluations and Data They Use

Comparative evaluations of LiDAR odometry report a narrow set of quantities. Among recent benchmarks, Będkowski et al. [13] evaluate twenty algorithms and Feng et al. [14] ten, both reporting trajectory error alone, and Ribeiro et al. [9] compare nine methods across four sensors, likewise evaluating accuracy alone, a restriction the authors state explicitly. Sier et al. [12] are the exception, recording CPU and memory occupancy and the rate at which poses are published, though not the time taken to process a scan. For a localisation system that has to run onboard, the relevant set of quantities is wider than accuracy alone.

The data on which these comparisons are run is carried almost entirely by ground vehicles and handheld rigs. Będkowski et al. [13] run two LiDAR sensors on a trolley along a laboratory rail and additionally evaluate on the third-party Bunker DVI dataset [35], Feng et al. [14] use a wheeled robot on a construction site, and Sier et al. [12] a wheeled platform, pushed or teleoperated, indoors and outdoors, and carried by hand through a forest. Ribeiro et al. [9] drive a wheeled omnidirectional robot through a laboratory hall with glass doors, a ramp, and an elevator, feeding its wheel odometry to three of the nine algorithms evaluated. Only Milijas et al. [15] use an aerial platform in their comparison, evaluating three LiDAR SLAM systems in a hangar, an agile-flight sequence, and around a campus building. Those systems predate the ones considered here, and the onboard computer is an x86 machine rather than an embedded one. None of these environments are a racked warehouse.

The reference against which the errors are computed varies with the setting. The public data on which LiDAR odometry is tuned carries references of survey grade, with poses registered against a prior map from a survey-grade laser scanner in Ramezani et al. [7], a total station tracking a prism, giving position without orientation, in Nguyen et al. [36] and, alongside motion capture, in Helmberger et al. [8], a satellite-aided inertial navigation unit in Geiger et al. [6], and motion capture in Ribeiro et al. [9]. Among the deployments of Section 2.2, the reference is thinner or absent, as Dias et al. [34] use motion capture only in a laboratory, Beul et al. [2] report GNSS in an outdoor sequence only, and in Kwon et al. [3] the reference is the pose derived from the same fiducial markers that correct the estimate. The closer a setting comes to the deployment considered here, the weaker the measurement that has been made in it.

### 2.4. Resource-Aware Evaluation on Embedded Platforms

The cost of running these systems is reported by few studies, and mostly outside of LiDAR odometry. In visual-inertial odometry benchmarks, Delmerico and Scaramuzza [17] measure pose accuracy together with per-frame processing time and CPU and memory load across four platforms, from a laptop down to an ARM single-board computer. Jeon et al. [18] repeat the exercise on three Jetson boards, reporting CPU and memory but not per-frame time, and Godio et al. [37] report CPU load for a visual-inertial pipeline flown in a warehouse on a Jetson Nano. On the LiDAR side, Sier et al. [12] measure CPU and memory utilisation and the rate at which poses are published across two ARM boards and two x86 machines. The rate, however, is obtained with the recordings being replayed at fifteen times real speed, so it measures how many poses a system can produce when pushed rather than whether it keeps pace with the sensor.

Where cost is left out, it is usually left out deliberately. Ribeiro et al. [9] state that their benchmark focuses exclusively on accuracy-related metrics, leaving out resource usage and real-time capability because they “depend strongly on the hardware platform and the runtime environment”, and describe only the containers that ran the systems, not the machine. The frameworks they point to as recording such metrics, SLAMBench [16,38] and SLAM Hive [39], report them for visual SLAM only. Elsewhere the hardware is simply absent, with Będkowski et al. [13] reporting trajectory error for twenty algorithms without characterising the machine, and Feng et al. [14] stating only that a unified hardware platform was used.

Measuring these quantities on the target is not the same as measuring them on a workstation. On embedded computers, sustained load typically raises the processor temperature until the clock is reduced, and throughput then depends on the thermal state and on the room. Benoit-Cattin et al. [40] report degradation within seconds under continuous inference and variation of up to 27.7% in throughput across an ambient range of 22 to 36 °C. Publication rate, the quantity usually reported, does not separate a system that keeps pace with the sensor from one that discards part of its input to stay current. A close case to the one considered here is the study by Gaona et al. [41], who evaluate LIO onboard a drone but report the rate at which poses are published rather than what producing them costs.

## 3. Materials and Methods

In this section, we describe the methodology used to conduct this benchmark. Section 3.1 states the pipeline used for setting up and conducting the benchmark. Section 3.2 describes the sensor, the aerial platform, and the two computing environments. Section 3.3 presents the scenarios used for this evaluation and the closed-loop flights. Section 3.4 details the algorithm tuning procedure and Section 3.5 explains how every measurement is done and how the evaluation is executed.

### 3.1. Benchmark Pipeline

The evaluation reports three quantities: the accuracy of the estimated trajectory, the time taken to process a scan, and the processor and memory occupied while doing so. They are not interchangeable and none substitutes for another. A system may be the cheapest per scan among those that work and still deliver its poses later than any other, and a per-scan time that appears affordable may be produced by a system that is discarding part of its input in order to keep pace. Each quantity is obtained by its own mechanism, and these are described separately in Section 3.5.

All six systems are evaluated by replaying recordings acquired in flight, at the acquisition rate of the LiDAR sensor, first on a workstation and afterwards on the embedded computer carried by the UAV, a protocol also adopted by comparable benchmarks [9]. The recordings, the configuration of each system, and the measurement tooling are identical on the two machines, so that any difference between them is attributable to the hardware alone. In this evaluation, the systems do not act on the platform at any point. Faster-LIO was afterwards flown in the vehicle’s control loop.

**Figure 2:**
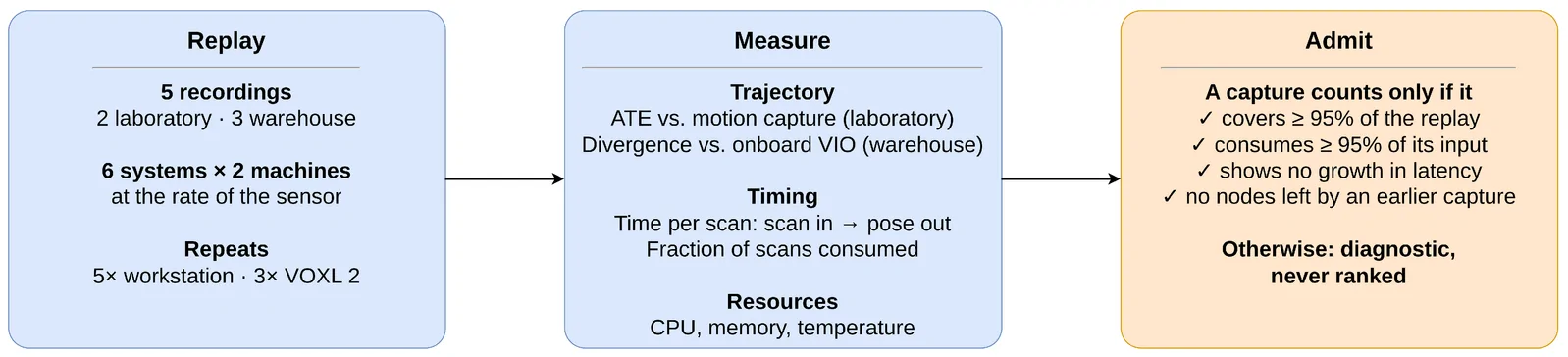
The path from a recorded flight to a line of a results table. One recording is replayed through all six systems in turn, on each of the two machines, and every combination of system, recording, machine and repeat is one capture. Each capture yields three independent measurements, and enters a results table only if all four acceptance criteria of Section 3.5.5 hold.

A single invocation of the harness replays one recording through all six systems in turn, repeating each system a fixed number of times, so that every figure entering a comparison was obtained under the same machine state. Each run is launched in isolation and produces three artefacts: the estimated trajectory, a timestamp for every input scan and every output pose taken on a single clock outside the system under test, and a sampling of the processor and memory occupied by the system’s own processes. A run becomes a result only once it satisfies a set of acceptance criteria, stated in Section 3.5, which establish that the system consumed the whole recording and the whole of its input, and that the machine was in a state comparable to that of the other runs it will be compared against. Scoring against the reference trajectory is performed afterwards, from the stored artefacts, and never during the run. Figure 2 summarises this path.

### 3.2. Hardware Used

The sensor used is a RoboSense Airy (RoboSense Technology Co., Ltd., Shenzhen, China), a mechanically rotating 96-channel unit turning at 600 rpm and delivering a point cloud at 10 Hz [42]. It has a 360° horizontal Field of View (FoV) and a vertical FoV spanning 0 to 90° upward from the horizontal plane, giving hemispherical coverage. It was operated in single-return mode.

**Figure 3:**
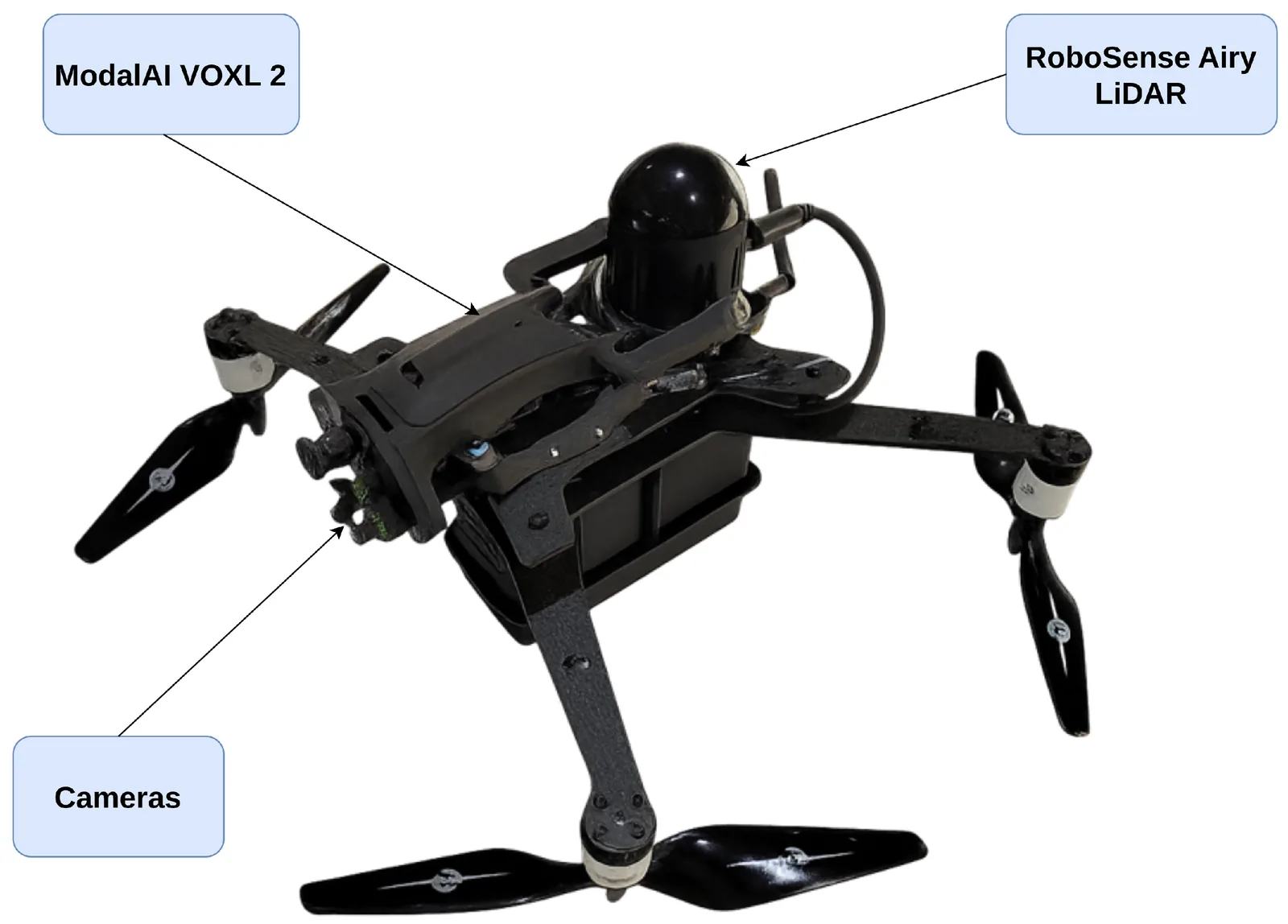
MANTIS UAV as flown in this work. The Airy LiDAR provides the input to the evaluated systems, the VOXL 2 runs them, and the tracking camera feeds the onboard VIO that serves as the reference at the warehouse.

The sensor is mounted above the aircraft, as shown in Figure 3, dome upwards with its *x* axis aligned with the vehicle’s forward direction, so that it observes the horizontal plane and the hemisphere above it. In a warehouse aisle, the geometry available to constrain the estimate is the rack structure above the flight altitude along with the ceiling, and the floor contributes no measurements. A warehouse floor is planar and close to featureless, whereas the racks carry structure, so the arrangement is deliberate.

Inertial measurements are taken from the six-axis unit integrated in the LiDAR, at its default rate of 200 Hz. Its raw output is expressed in multiples of *g* and is converted to m/s^2^ before reaching the systems under test, outside the process tree over which computation is measured. The inertial and ranging origins coincide, and the inertial axes are rotated by 180° about the *x* axis with respect to the point cloud frame, confirmed against gravity at rest and the sign of the reference altitude.

The recordings were acquired with the MANTIS UAV, a vehicle developed at INESC TEC (Porto, Portugal) for inventory operations in warehouses [34], carrying the Airy above its airframe for this work and flying under a PX4 autopilot. The platform can be seen in Figure 3. This drone runs a visual-inertial estimator on a fisheye tracking camera and an inertial unit of its own, with the estimate serving as the trajectory reference at the site where motion capture is unavailable, as described in Section 3.3. The estimator has been developed further since the platform was published, so the figure reported there does not describe the configuration flown here. It was characterised instead on the two laboratory recordings of this study, in which motion capture and the visual-inertial estimate were recorded at the same time, and its error against motion capture is 0.10 m in Root Mean Square Error (RMSE) and 0.24 m at maximum, agreeing between the two recordings to within a millimetre. The vehicle’s onboard computer is a ModalAI VOXL 2 (ModalAI, Inc., San Diego, CA, USA), which is also the embedded target of this evaluation.

Two machines are used, and every cost figure reported in this work belongs to exactly one of them. The first is a mobile workstation, an HP EliteBook 640 G9 (HP Inc., Palo Alto, CA, USA) with an Intel Core i7-1270P (Intel Corporation, Santa Clara, CA, USA) of twelve cores and sixteen threads, and 32 GB of memory, running Ubuntu 24.04 and ROS Noetic inside a distrobox container running Ubuntu 20.04. The second is the vehicle’s own computer, a ModalAI VOXL 2 built on a Qualcomm QRB5165 (Qualcomm Technologies, Inc., San Diego, CA, USA), whose eight cores comprise four Cortex-A55 at 1.805 GHz, three Cortex-A77 at 2.419 GHz and one Cortex-A77 at 2.842 GHz, with 7.7 GB of memory and the same ROS distribution and container arrangement [43]. The evaluated systems were pinned to cores 4 to 7, which correspond to the four Cortex-A77, setting a ceiling of 400% CPU consumption. Processor frequency is not reported, as the CPU is set to performance mode for the whole evaluation, setting the frequency to its maximum operating value. The vehicle’s flight services were left running for the whole of the evaluation, occupying a measured 1.9 cores. Since the vehicle cannot fly without those services, this is the operating point at which these systems would have to run, and the cost measured here is contention-inclusive by design rather than by oversight.

### 3.3. Scenarios

Five recordings were acquired at two sites and are summarised in Table 2. Every parameter of every system was selected on the two laboratory recordings, and the three warehouse recordings were held out and took no part in parameter tuning. This division is a property of the design rather than of the analysis, and it is what allows the warehouse results to be read as evidence rather than as confirmation.

**Figure 4:**
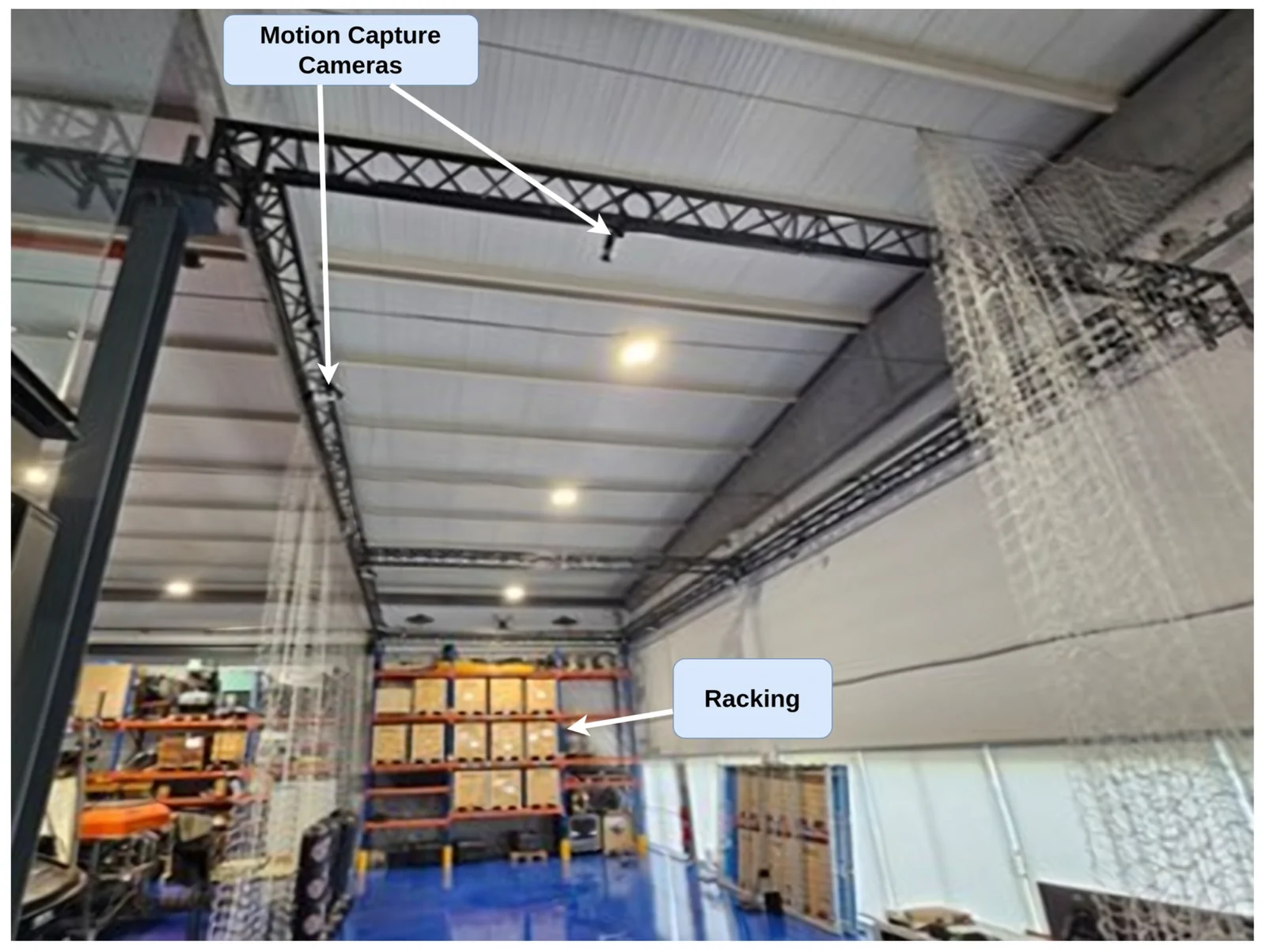
The laboratory site, where racking runs along one side only and the twelve-camera motion capture system provides the reference for the two recordings of the benchmark and the eight closed-loop flights.

The laboratory, shown in Figure 4, is instrumented with a twelve-camera Qualisys motion capture system (Qualisys AB, Gothenburg, Sweden), which provides the reference at that site. It reports the drone position at about 100 Hz, and over segments in which the drone rests on the ground that position varies by less than 3 mm, two orders of magnitude below the smallest trajectory error reported in this work. Marker occlusions interrupt the position output from the motion capture. Short interruptions occur throughout both recordings, and in the picking trajectory the drone leaves the tracked region altogether for 24.7 s, one of four interruptions that total 28.4 s. Estimated poses falling inside these interruptions have no reference to be compared with and are excluded rather than interpolated. This site has two flights: the first one is a square, where the drone sets its altitude to 1.5 m and then flies a 2 × 2 m square, yawing towards its direction of travel; the second one is a picking trajectory, where the drone flies laterally facing the warehouse racks, simulating a stock picking trajectory, travelling along the *y* and *z* axes.

The warehouse is an operational logistics facility at the Volkswagen Autoeuropa plant, and no metric reference is available in it. The recordings made there carry the vehicle’s own visual-inertial estimate instead, whose uncertainty, characterised in Section 3.2, is of the same order as the trajectory errors that would be scored against it; the laboratory volume in which it was characterised is also smaller than the distances flown here, so that figure is a lower bound at this site. These recordings therefore establish whether a system continues to operate at a site it was not tuned on, and cannot rank the accuracy of those that do. At this site, three recordings were made: on the first recording, the drone follows a 2 × 5 m rectangle at 1.5 m of altitude, a flight similar to the first one at the laboratory; the second flight follows a stock picking trajectory on both sides of the aisle, with motion along every axis and rotation in yaw; the last recording is a flight over the whole aisle, on which the drone flies to the end and back to its starting point and additionally performs a stock-picking manoeuvre before landing. This last recording is the only one without a localisation reference, as this was a manual flight. It is nevertheless included to evaluate processor and memory consumption, since it is the longest trajectory.

Eight further flights were made at the laboratory, and none of them take part in the benchmark. In these, Faster-LIO ran on the vehicle’s computer and supplied the position to its PX4 autopilot, so the estimate was inside the control loop and not replayed from a recording afterwards. The visual-inertial estimator ran alongside without entering the control loop, and motion capture observed the flights without feeding anything back, so both estimates are scored against an independent reference within the same flight. Four manoeuvres were each flown twice, as described in Table 3. Only pose was logged, so these flights carry no processor, memory or temperature figure.

### 3.4. System Configuration and Tuning

A package name and a citation do not identify what was run. Each system was built from a specific revision of its public repository, listed in Table 4, and each required at least a sensor port and a configuration and launch pair. Four carry modified C++. The largest of those modifications is the Airy driver port in the preprocessing stage of FAST-LIO2, without which the sensor’s point type is not read at all.

Two systems diverge from the description a reader would obtain from their citations, and Section 2.1.3 defers both to here. The F-LOAM implementation evaluated, built from revision 20062e3 of its public repository, applies neither the two-stage distortion compensation nor the adaptive feature weighting proposed in the paper. The relative timestamp of each point is computed and carried in the intensity field, but no stage of the odometry reads it, so the two-stage distortion compensation is not performed; and the residuals are formed with no weighting term, local curvature serving to select edge and planar points but never to weight them. What is evaluated is the feature-based scan-to-map registration the implementation performs, and it is to that pipeline, rather than to the method as published, that the results refer. liorf, in turn, removes the feature extraction stage of LIO-SAM rather than replacing it: every deskewed point becomes a candidate planar point, and the residual is formed against a plane fitted at match time to the nearest map neighbours. What this changes is that no edge residual exists. What it does not change is the residual family, which remains point-to-plane exactly as in FAST-LIO2, so removing feature extraction does not make the system featureless in the sense of KISS-ICP, which carries no surface model at all.

Configuration then proceeded in two steps, and the order between them matters. The systems were first brought onto common ground, because they were not being shown the same scene: the range beyond which each discards returns differed by a factor of two across the set, in an arena no larger than 15 m. Minimum and maximum range were equalised to 0.45 m and 15.0 m wherever the parameter exists. This is a result rather than housekeeping, since the range clip alone moved KISS-ICP on the laboratory square from 0.763 m of trajectory error to 0.033 m: a comparison of default configurations is in part a comparison of field of view. Range was equalised before any other parameter was selected, because tuning at one range and changing it afterwards invalidates the tuning.

With the input equalised, one structural parameter was then swept for each system, on both laboratory recordings, at three repetitions per value, and with the optimum bracketed rather than assumed. For the two systems that share an estimator, every parameter they read under the same name was swept on both, so that the axis on which they are compared is not itself a tuning difference. Two of the sweeps returned negative results that are worth stating: the iteration count is inert between one and four on both filter systems, a result predicted from the early exit in their shared source and only then measured; and the map resolution of F-LOAM was swept and left at its default. Two systems reached their best accuracy at a voxel coarser than their upstream default, and for the same reason in both cases: those defaults target sensors of sixteen to thirty-two beams, whereas the Airy places ninety-six at short range and is considerably denser per unit area, so a coarser leaf is the expected direction rather than a surprise.

The noise characteristics of the inertial unit were obtained by Allan variance over approximately 24 h of static logging. The resulting noise densities are used directly by liorf, which is parameterised in those terms. The two filter systems are parameterised by covariance instead and retain values obtained by search, because the converted static figures cause divergence on these recordings, as does every value below about $10^{-1}$. The reason is physical rather than numerical: in flight the accelerometer is dominated by airframe vibration rather than by sensor noise, with a standard deviation above the cloud rate exceeding 10 m/s^2^, some three orders of magnitude above the static characterisation. A noise model obtained from a stationary unit is not transferable to the same unit in flight. The shipped values were confirmed by a sweep spanning six decades in both terms, within which no value improved on them by more than the run-to-run spread. DLIO estimates its inertial biases at initialisation and exposes no equivalent parameter.

Two properties resisted equalisation and are stated rather than patched. Point decimation is a property of each system’s own pipeline: two consume every fifth point and one every third, while three consume every point, so the pair that shares an estimator is a like-for-like comparison whereas the wider set is not. And DLIO exposes no maximum range parameter, so it alone could not be brought onto the common range limit.

The protocol changes the conclusion, which is the argument for adopting it. Under unequal tuning effort the comparison favours one system; under equal effort on the parameters two of them share, neither dominates; and with each system at the best configuration found for it, a third ordering emerges. Two publishable conclusions were produced by tuning effort alone before the third was produced by the data.

### 3.5. Measurement and Evaluation

#### 3.5.1. Accuracy

Each estimate is first transformed into the body frame of the reference, using the frame in which that particular system publishes its poses, which is recorded for every run. This step is easy to omit and expensive to omit: scoring a trajectory expressed in the sensor frame as though it were expressed in the inertial frame moves the position error by less than a millimetre, while placing the rotation error 180° away from its true value. Rotation is the only quantity that reveals the mistake.

Estimated and reference poses are then associated by nearest timestamp within 20 ms—under two centimetres of vehicle motion at the highest speed flown, and under two millimetres at the median—and pairs that straddle an interruption of the reference are rejected. Two alignments are applied to the associated pairs and both are reported. The first is a rigid alignment in SE(3) obtained by least squares over the whole trajectory, which is what comparable studies report; scale is not fitted, because range from a LiDAR is metric and an error in scale would be a real error rather than a gauge freedom. The second aligns the two trajectories at their first pose alone, which removes the arbitrary choice of world frame and nothing else. The two are not redundant. A least-squares fit over the whole trajectory will rotate the estimate in order to reduce the sum of squared position residuals, and in doing so absorbs exactly the accumulated heading error that a corridor traverse produces; the difference between the two figures is therefore a measure of how much heading error the first of them concealed.

Alongside them, the rotation and yaw errors are reported under the origin alignment, and the relative pose error is computed over one-second intervals. The relative measure requires no alignment convention at all, since it uses only the increment between two poses of the same trajectory, and it is on this measure that the claims about drift rest. Pairs spanning an interruption of the reference are skipped. No run is excluded on the basis of its error. A run is admitted or rejected by the four criteria of Section 3.5.5 alone, and its error is then reported as measured.

The interruptions of the laboratory reference are themselves informative. Across each of them the vehicle continues to fly with no reference at all, so the change in relative motion between the last pose before the interruption and the first pose after it measures unaided drift directly, over an interval in which nothing corrects it. This quantity is reported for every interruption, in position and in rotation, and, like the relative measure, requires no alignment convention. All of the measures above require a metric reference and are computed at the laboratory alone. At the warehouse, where the available reference is itself an estimate, what is reported is divergence from it under the same relative measures, and no absolute error is claimed.

#### 3.5.2. Computation

The times the systems report about themselves are not used, because they are not measurements of the same thing. One prints a running average to standard output; one retains the duration of the most recent scan only; one accumulates timings into a structure that is never written out; one records them behind a compile-time flag that is disabled by default; and two report nothing at all. Each defines its own start and end point within its own pipeline. Comparing these figures across systems would compare their authors’ instrumentation rather than their cost.

Cost is therefore measured off the wire, identically for every system. A single recorder process subscribes to the point clouds entering the system and to the poses leaving it, and timestamps both arrivals on one monotonic clock, so that the difference between them is the wall-clock interval that system took to turn that scan into a pose. The point clouds are subscribed as opaque messages and never deserialised, with a queue long enough that the recorder itself never discards one, so that the 2 MB of payload per scan are never paid for by the instrument; only the message header is unpacked, to read the stamp. The recorder’s own hop between the two subscriptions is below a millisecond and is not subtracted. What this yields is an end-to-end figure. It includes preprocessing, the state update, publication, and any interval the scan spent waiting behind a busy thread, which is the quantity that determines whether a system keeps pace on the vehicle. It is not an isolated kernel time, and Section 3.5.3 sets out the ways in which reading it as one is wrong.

Matching a pose back to the scan that produced it is the one delicate step, because the systems do not agree on what the timestamp of an odometry message denotes: four of them copy the stamp of the scan, while the two that share an estimator stamp with the end of the sweep, one scan period later. At 10 Hz that offset equals the interval between scans exactly, so a pose lies equidistant between its own scan and the following one and nearest-neighbour matching is genuinely ambiguous. The offset is therefore measured for each run rather than assumed: every difference between a pose stamp and a scan stamp within ±0.15 s is accumulated into a histogram, the peaks of which are the candidate offsets, and candidates are eliminated by causality, since a pose cannot be published before the scan that produced it. The smallest surviving candidate is used, and the value chosen is recorded with the run.

#### 3.5.3. Four Ways a Per-Scan Latency Misleads

The interval measured in Section 3.5.2 is what a system costs end to end, and it is the figure that determines whether the sensor can be kept up with. It is not, however, the cost of the computation, and treating it as such fails in four distinct ways. Each was found by measurement rather than anticipated, and each is corrected in the tooling. Two quantities are therefore reported for every run: the end-to-end interval, which is the control, and the same interval less the buffering that is structural to the system, which is the figure that answers whether the computation would fit on the vehicle. For one of the six systems these two differ by a factor of four.

The first failure is structural input buffering read as computation. One of the evaluated systems caches two scans before it begins work at all, so that roughly two scan periods of its end-to-end interval are spent waiting for input that has not yet arrived. Read as computation, its cost is overstated by a factor of four, and it is ranked last among the systems that work when it is in fact cheaper per scan than another that is ranked above it. Buffering is distinguishable from overload by its shape: a structural buffer is a flat floor, present in full on the very first pose, whereas a system falling behind accumulates. It was confirmed causally rather than inferred, by reducing the cache from two scans to one and then to none, which moved the median interval from 263 ms to 170 ms and then to 69 ms with no scan discarded, each step removing approximately one scan period.

The second failure is unbounded queueing. A system that runs over budget and does not discard input accumulates a queue without limit, and its end-to-end interval then measures the depth of that queue rather than the work: one of the systems evaluated reached a median interval of 26.9 s for scans arriving every 100 ms. The interval is real, and it is the correct answer to whether the system keeps pace, but it is not the cost of a scan. That cost is recoverable from the rate at which the system falls behind. If *P* is the interval between scans and *r* the rate at which latency accumulates per second of replay, then the time taken per scan is

(1) C=P(1+r).

Applied to the run above, Equation (1) recovers 137.2 ms, against the 133.9 ms that system prints for itself, which is the one case in which a self-reported figure is available for comparison.

The third failure is scoring throughput against a system’s own output. Dividing the interval by the rate at which a system publishes poses measures whether it is self-consistent, not whether it keeps pace with the sensor. A system discarding two scans in every three scores 0.82 by that measure and reads as comfortable; against the period at which scans actually arrive, the same run scores 2.23. Any rate is therefore reported together with the fraction of the input the system actually consumed.

The fourth failure is that truncation is invisible from the output alone. Every quantity derived from what a system publishes remains nominal on a run that stopped early: the rate is steady, there are no gaps, the fraction of consumed input is unity, and the trajectory is simply shorter. One system lost the final 89 s of a recording, 26% of its duration, and was scored against the reference as though it had completed. Only a check of the span actually covered against the duration of the replay detects this, and no run is admitted as a result until it passes one.

#### 3.5.4. Processor, Memory and Temperature

The processor and memory occupied by each system are sampled at 2 Hz by a monitor that runs inside the same launch as the system under test and takes as its subjects the child processes of that launch. The filtering is therefore structural rather than a list of names: the replay, the trajectory recorder and the inertial unit conversion are started elsewhere, are not children of that launch, and can never enter the total. One support node is an exception, being forked by the same launch as the system itself, and it cannot be excluded by executable name because every node written in Python 3.8.10 reports under the same interpreter name; it is excluded instead by its ROS node name, which the launch appends to the command line of every node it starts. The distinction is not academic: before it was drawn, that node contributed roughly 48 MB and 2% of a core to what was reported as the cost of one of the evaluated systems. Both the process name and the ROS node name are recorded with every sample, so that what was counted is auditable rather than implied by a total.

On the embedded platform the same role is filled by a native implementation sampling at the same rate, which additionally records the occupancy of each core and the temperature of the processor. Temperature is read from the sensor of the cluster on which the systems run; the two general-purpose processor sensors understate the same load by approximately 11 °C and are not used. Processor frequency is not reported, as the governor is fixed to performance for the whole of the evaluation and holds every core at its maximum operating point. Any thermal limitation is therefore not visible as a reduction in the reported clock rate.

#### 3.5.5. Execution Protocol

Each recording is replayed at the rate at which its scans were acquired. The simulated clock is published at a finer granularity than the replayer’s default, whose 10 ms period would quantise the very intervals being measured. All six systems are replayed within a single invocation for each recording, which is a control rather than a convenience: it is the only arrangement in which every system meets the same machine, so it is the only comparison in which the state of that machine is shared rather than assumed to be equal.

Replay is not deterministic. Two replays of the same recording through the same system differ by approximately 1.4 cm on average and 4.8 cm at worst. Every figure reported in this work is consequently a median over repeated runs, quoted with the range observed, and a difference smaller than that spread is not treated as a difference. Runs are repeated five times on the workstation and three times on the embedded platform. Five rather than three because one system is bimodal on one of the laboratory recordings, taking one of exactly two trajectory errors according to a single scan at start-up; with three repeats the median is decided by which of the two happens to occur twice.

The state of the machine is itself controlled. A fixed integer workload is timed before every capture and its result recorded alongside the run, and a capture whose result departs far from the established healthy reference is not used. This exists because the workstation was found to lose a factor of two to three in sustained throughput over the course of a long session, recoverable only by rebooting, with no thermal cause: the processor was at 38 °C, the throttling counters were at zero, and the machine was on mains power at full performance. Everything measured before this control was in place is excluded from the results. The control has a known limit on the embedded platform, where the benchmark is necessarily sampled before the system launches, which on a passively cooled device is a state the workload never subsequently runs in; costs of different systems are therefore compared only within one session.

A run is finally not admitted as a result until it satisfies four criteria. The span actually covered by the estimate is checked against the duration of the replay, and must reach at least 95% of it, which detects truncation. Only a span that ends early counts as truncation. DLIO withholds its output until the vehicle first moves, and a span that begins late is the consequence of that start-up gate. The fraction of input scans the system consumed is checked, and must also reach 95%, which detects silent discarding. The rate at which latency accumulates is checked, which detects queueing. And the process table is checked for nodes left behind by an earlier capture before each run begins. This last criterion exists because of a defect in the harness. The launcher starts each node in its own process group, so signalling the launcher’s group never reached them; when the launcher exceeded its grace period and was killed outright, its nodes were orphaned. One survived for 8.4 h, still registered under the same name and still subscribed to the point cloud topic, colliding with every capture that followed and inflating timings by 3–19% non-uniformly. It was corrected by enumerating and reaping descendants explicitly, and the affected measurements are among those excluded above.

The first three criteria are not interchangeable, and the reason is the substance of Section 3.5.3. A system that queues announces itself on three of them at once: it consumes every scan, its latency climbs steadily, and its memory grows with the queue. A system that discards input announces itself on one. In the runs reported here, the system that discarded 57% of its scans held its latency flat, covered 99.5% of the recording and occupied 76 MB, against the 1.18 GB of the system that queued. An evaluation reporting latency, coverage and memory alone would rank the discarding system as the healthier of the two.

## 4. Results

This section reports the results of the evaluation, and is divided by platform and site. Section 4.1 and Section 4.2 report the laboratory and warehouse results on the workstation, and Section 4.3 and Section 4.4 report the same on the embedded platform. Section 4.5 then compares the selected system against the estimator the vehicle already carries, and Section 4.6 reports it flown in closed loop.

### 4.1. Desktop—Laboratory

**Figure 5:**
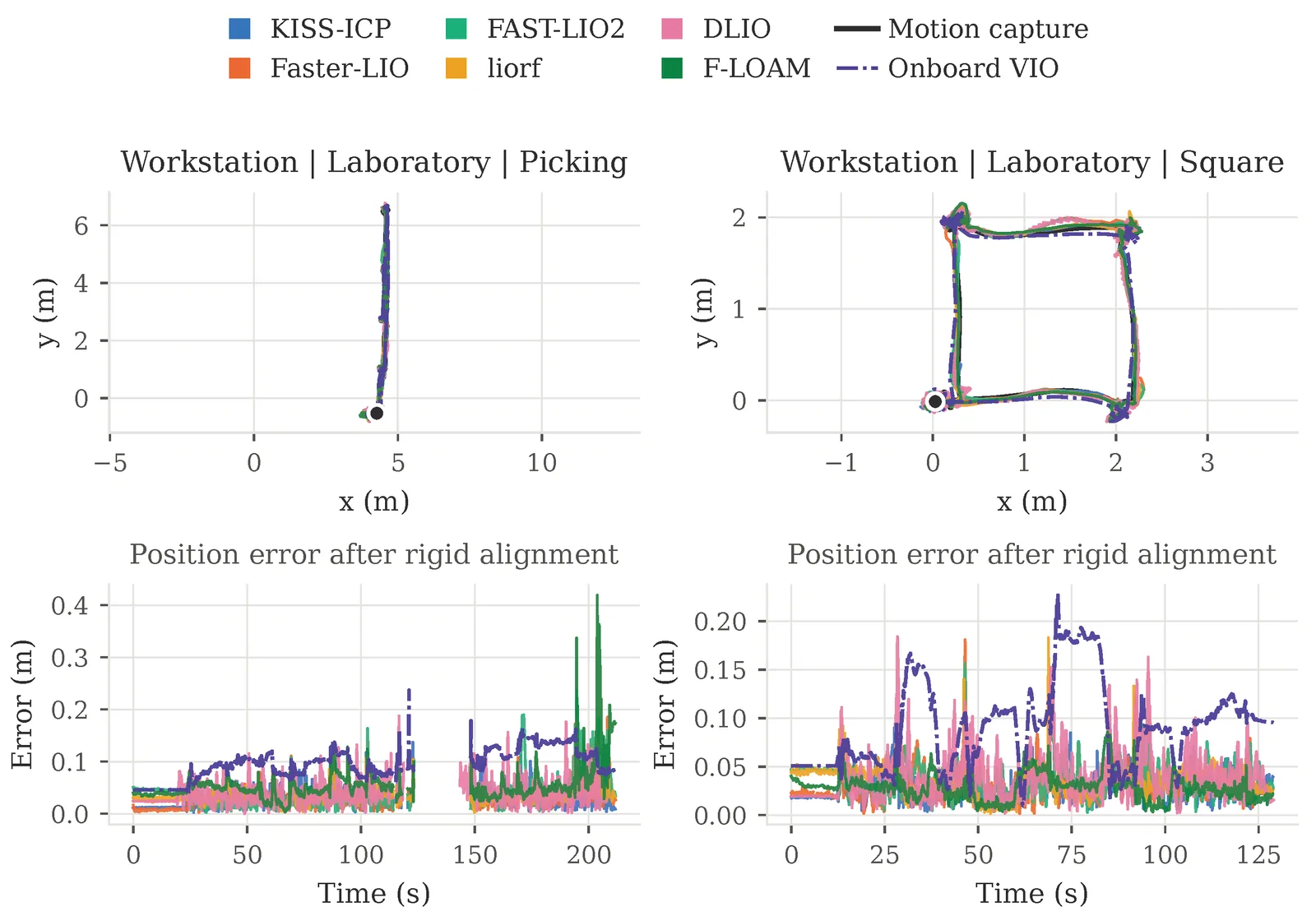
Estimated trajectories at the laboratory site on the workstation, against the motion capture reference, with the vehicle’s own visual-inertial estimate drawn for comparison and the per-pose error on a shared time axis. The estimates overlie one another and the reference at this scale, and in error all of them stay below the visual-inertial estimate over most of both recordings. The interruption in the picking recording is a dropout of the reference, across which no error is computed. The black marker indicates the start of each trajectory.

Table 5 reports the six systems at the laboratory site at each configuration of Table 4, and Figure 5 shows the paths they estimate. All six keep pace with the sensor, consuming between 99% and 100% of the input scans, and none are close to the 100 ms budget.

Accuracy separates them by less than a factor of two. Every system lands between 0.031 m and 0.066 m across both recordings, and the five repeats of each agree to within 1.5 mm in all but two cases. The first is FAST-LIO2 on the picking trajectory, which returns one of exactly two values, 0.047 m or 0.051 m, according to a single scan at start-up, the bimodality for which five repeats instead of three were used. The second is liorf on the same recording, whose repeats span 0.038 to 0.042 m. The most accurate system on both recordings is KISS-ICP, which uses no inertial data, and it is also the cheapest in memory at 62–63 MB.

Cost separates the systems considerably. The two filter-based systems complete a scan in 6–7 ms against 39–43 ms for F-LOAM, a factor of six, and Faster-LIO occupies 0.38 of a core against the 1.4–1.5 cores of KISS-ICP. The Pareto front on this machine therefore has two members: KISS-ICP, which is the most accurate and the cheapest in memory, and Faster-LIO, which is the cheapest in CPU, third in accuracy by less than five millimetres, and near-lowest in memory.

One system resists summary. F-LOAM is second of six on the square and last of six on the picking trajectory, with no configuration difference between the two, and its cost is the highest at both. Its result is that variability, and a single figure quoted for it would misrepresent it in either direction.

### 4.2. Desktop—Warehouse

No parameter of any system was selected on the warehouse recordings, so Table 6 reports what the laboratory configurations do on data they were not fitted to. On the workstation, all six keep pace again, consuming 99% to 100% of the input scans.

Five of the six agree to within a centimetre of one another on the picking trajectory, between 0.141 m and 0.149 m, and all six agree to within three centimetres on the rectangle, between 0.104 m and 0.133 m. Those figures are at or within the uncertainty of the reference itself, characterised in Section 3.2, and they cannot be ordered.

**Figure 6:**
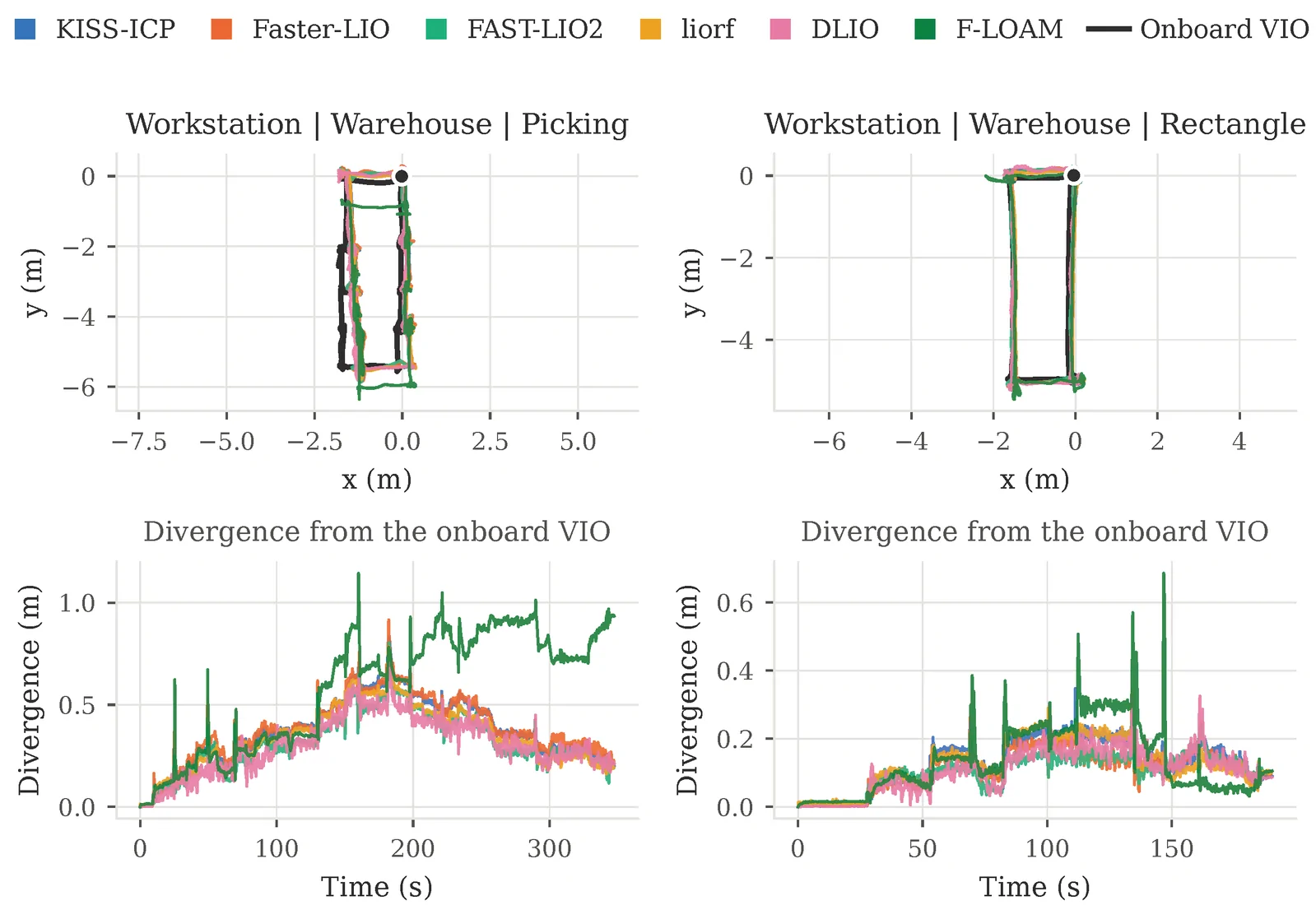
Estimated trajectories at the warehouse site on the workstation, against the vehicle’s own visual-inertial estimate, with the per-pose divergence on a shared time axis. F-LOAM separates from the others part-way through the picking trajectory and does not recover; the remaining systems stay within a narrow band of one another throughout both recordings. The divergence plotted here is origin-aligned and is therefore larger than the rigidly aligned figures of Table 6. The black marker indicates the start of each trajectory.

F-LOAM is the one system that does not transfer, and Figure 6 shows it. Its divergence from the reference on the picking trajectory is 0.346 m, more than twice that of the system with the next-highest divergence, and its cost rises from the 39–43 ms of the laboratory to 58–79 ms. The rise is the map growing. F-LOAM rebuilds two *k*-d trees over its local map on every scan and crops that map only beyond 100 m of the current pose, a bound no trajectory in this study approaches, so the map grows with the area explored. On the picking trajectory its time per scan climbs from 53 ms over the first tenth of the replay to 94 ms over the last, while Faster-LIO moves by under two milliseconds over the same span. The accuracy has no such explanation. The implementation compensates no motion distortion (Section 3.4), which is a plausible contributor to the 0.346 m but not an established cause, since these recordings are dominated by hovering.

**Figure 7:**
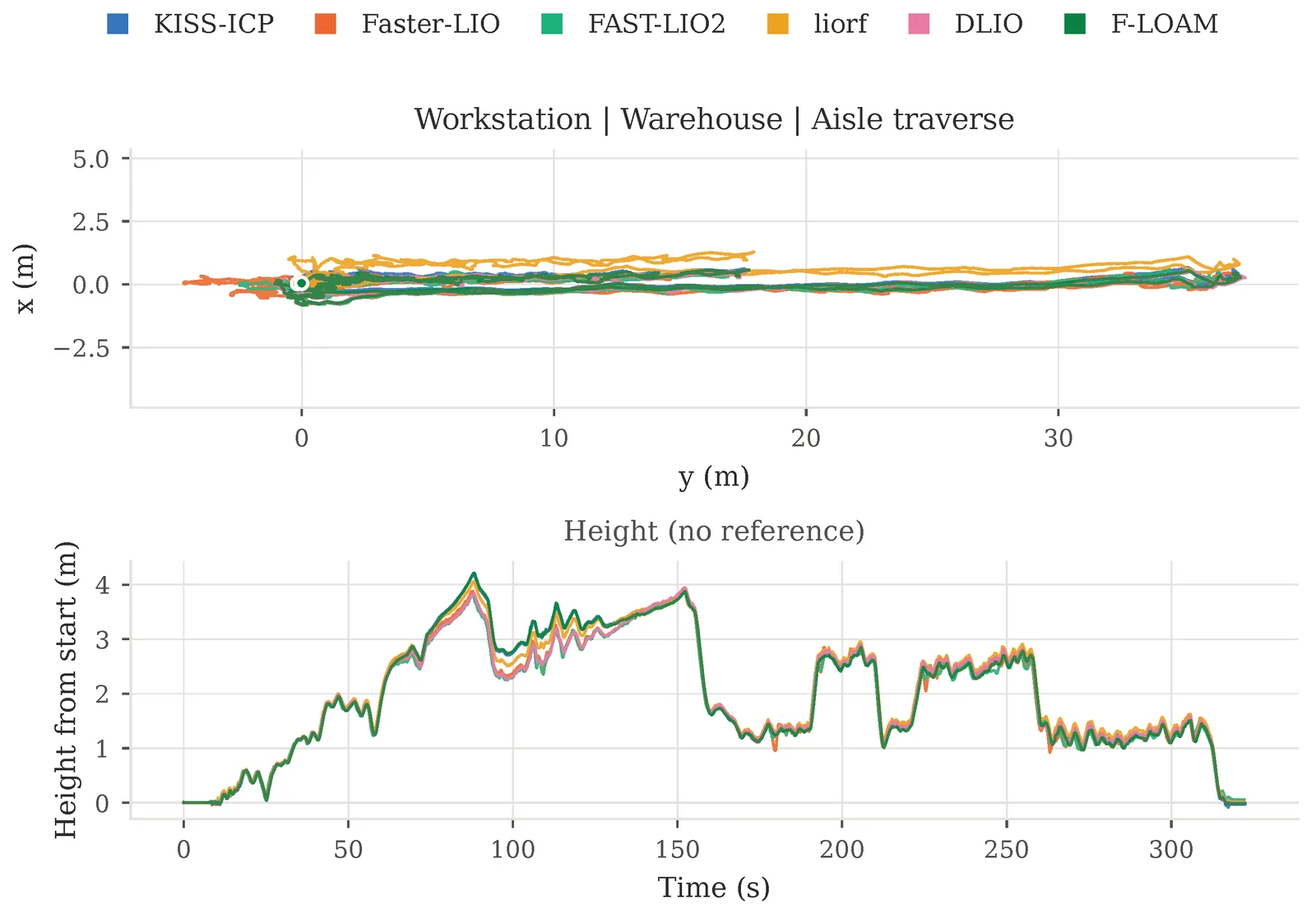
The six estimated trajectories on the aisle traverse, seen from above and in height over time. No reference exists for this recording, so nothing here is a measurement of accuracy and the recording contributes cost alone. Each package works in its own frame, and the sign conventions have been brought to a common one. Path length is not comparable across all six, because DLIO propagates between scans and returns about ten poses for every one the others return. The white circle marks the start of each trajectory.

The six estimates on the aisle traverse are shown in Figure 7. With no reference for that recording, they can be compared with one another but not scored.

### 4.3. Embedded—Laboratory

The same recordings and the same configurations were then replayed on the vehicle’s own computer, with the systems confined to its four large cores. Table 7 reports the laboratory site on that platform, from a session in which all five recordings were replayed in one sitting and therefore share a single thermal history.

**Figure 8:**
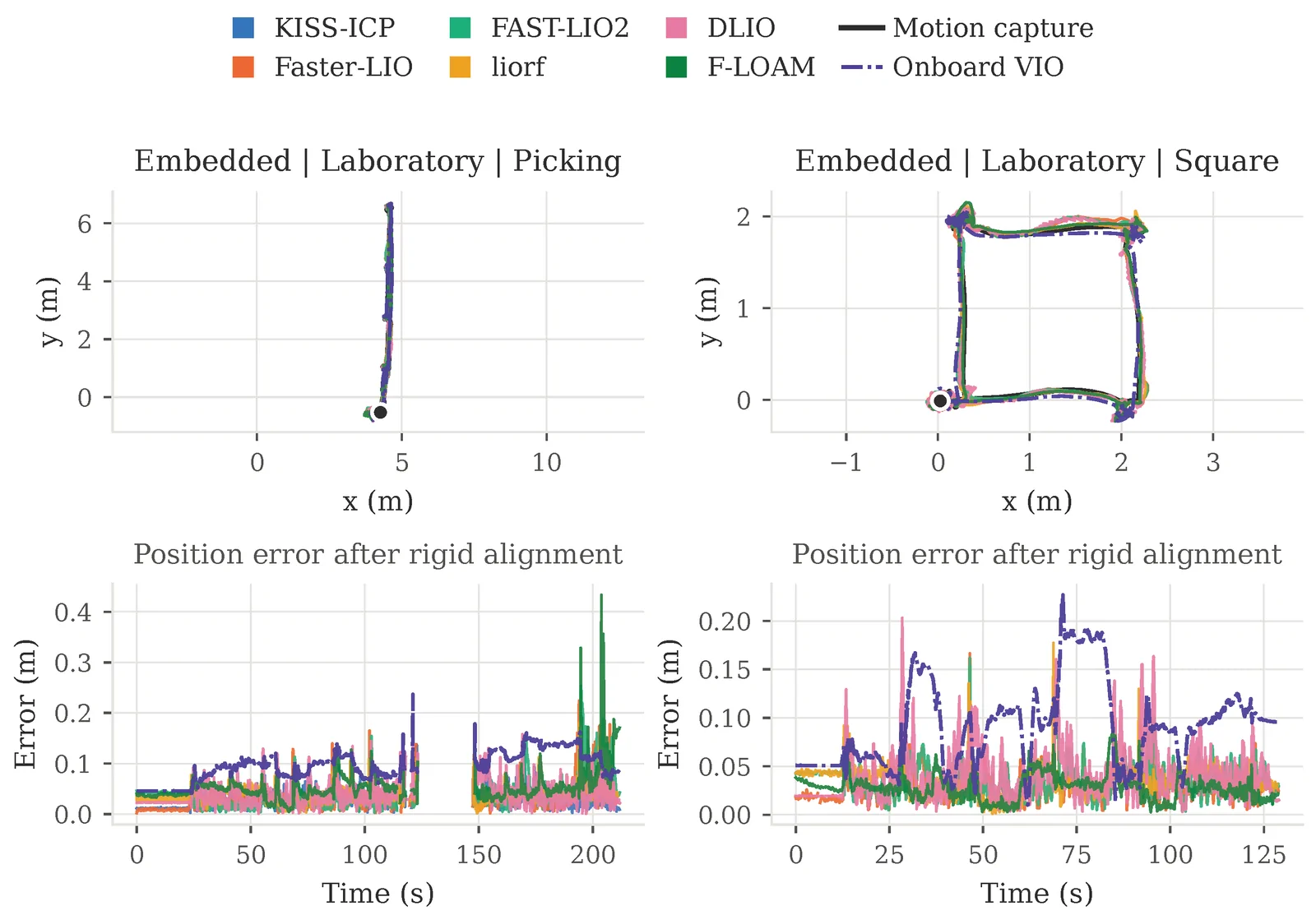
The same laboratory recordings and the same reference on the embedded platform. The figure is indistinguishable by eye from Figure 5, which is the accuracy-transfer result of this section stated visually: between the two machines, what changes is the cost, not the trajectory. KISS-ICP is absent from the square, the one capture at this site that did not consume its input. The black marker indicates the start of each trajectory.

Accuracy transfers essentially unchanged, and Figure 8 states it before the table does: placed beside its workstation counterpart, it is the same picture. Ten of the twelve laboratory figures fall within about 5% of their workstation counterparts, and the changes of rank among them are between pairs separated on the workstation by less than a millimetre, which is to say between systems that were already indistinguishable. Of the other two, DLIO on the picking trajectory differs by 3 mm, below the spread between replays, and KISS-ICP on the square is 18% worse, and it is the one system that did not consume its input.

Cost does not transfer, and it does not transfer uniformly. The penalty is smallest for the systems that do their work in one thread and largest for those that distribute it. In this session, F-LOAM slows by a factor of roughly 1.8, liorf by 2.7, and the two filter-based systems by close to 3.9, and DLIO, replayed in a later session together with Faster-LIO, slows by 1.8 against 3.2 to 3.6 for Faster-LIO in that same session. The division is not by cost, nor by modality, nor by estimator family; it is by how much of the work each implementation spreads across threads, and the systems that lose the most are those that had the most threads to lose when twelve cores became four. The ordering is the durable result and the factors are not: in an earlier session with the board 20 °C cooler the same measurements gave 1.6 to 3.4 rather than 1.8 to 3.9.

**Table 7:** The six systems at the laboratory site on the embedded platform, over three repeats, confined to the four large cores. Trajectory error is the RMSE after rigid alignment, taken as the median across repeats. Peak temperature is that of the large-core cluster, median across repeats. In each recording, bold marks the lowest trajectory error, time per scan, CPU and memory among the rows admitted as results, and, in the scans-consumed column, captures that consumed less than 95% of their input.

System	Absolute Trajectory Error (m)	Time per Scan (ms)	Scans Consumed	CPU (Cores)	Peak RSS (MB)	Peak Temp. (°C)
*Square*						
F-LOAM	**0.0312**	79.4	1.00	0.96	105	93.5
KISS-ICP	0.0371	105.2 ^1^	**0.80**	2.46 ^1^	51 ^1^	96.2 ^1^
FAST-LIO2	0.0406	26.8	1.00	**0.30**	196	74.7
Faster-LIO	0.0374	**24.0**	1.00	0.38	71	72.0
liorf	0.0384	34.5 ^2^	1.00	0.76	124	79.4
DLIO	0.0471	35.5	1.00	0.52	**55**	74.3 ^3^
*Picking*						
F-LOAM	0.0643	70.2	1.00	0.88	105	86.0
KISS-ICP	**0.0349**	59.9	0.97	1.92	**51**	95.4
FAST-LIO2	0.0492	**23.4**	1.00	**0.28**	192	70.8
Faster-LIO	0.0403	25.2	1.00	0.38	74	75.1
liorf	0.0411	35.5 ^2^	1.00	0.76	133	82.1
DLIO	0.0459	34.4	1.00	0.52	58	75.5 ^3^

^1^ Exceeds the 100 ms budget in all three repeats, consuming 80% of the input, so no capture of this system on this recording met the acceptance criteria of Section 3.5.5; the row characterises the failure and is not a result. In the earlier session this system held the budget at 51 ms (Figure 9). ^2^ Computation, with the two scans of structural input buffering removed; see Section 3.5.3. ^3^ Replayed in a later session together with Faster-LIO, so its cost does not share the thermal state of the other rows.

**Figure 9:**
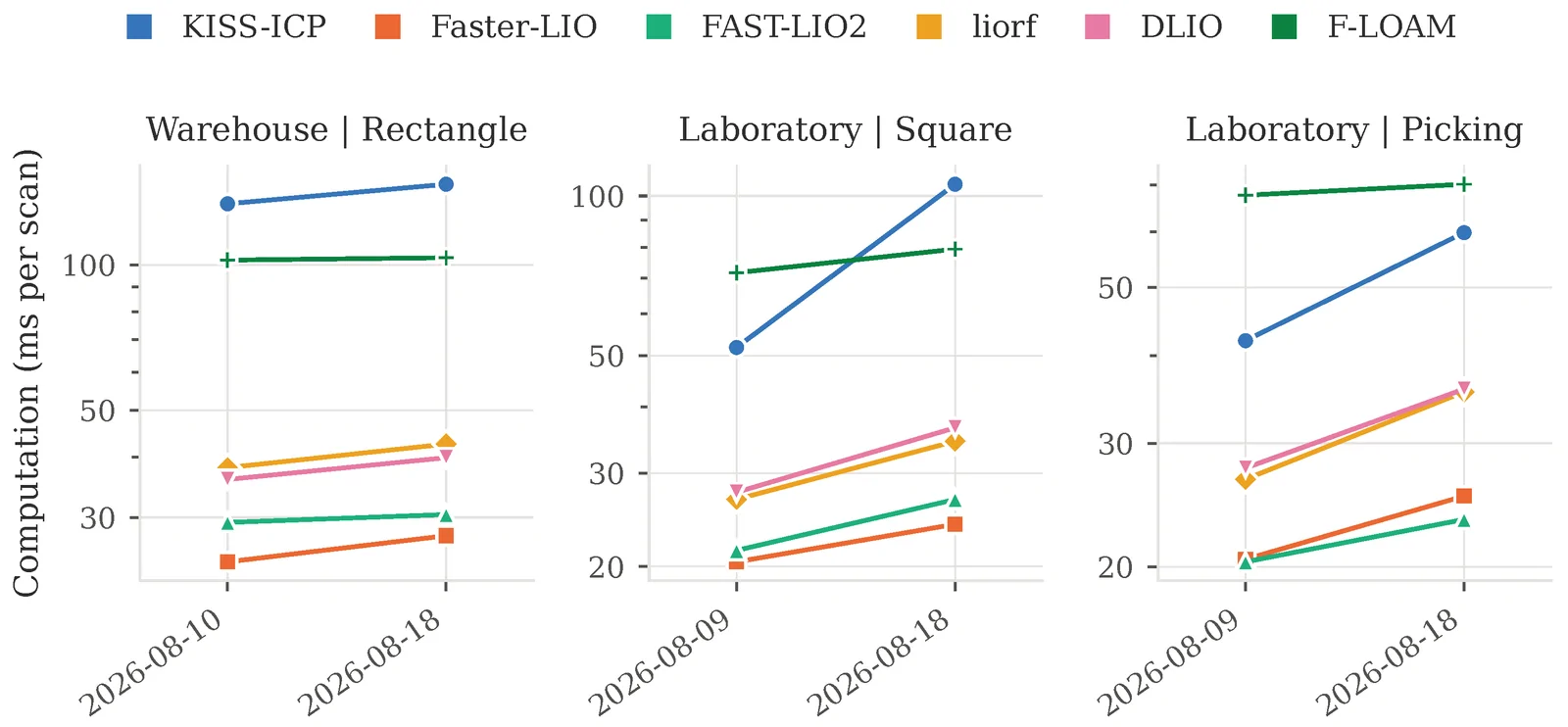
Computation per scan for the same recordings on the embedded platform in two sessions, nine days apart at the laboratory and eight at the warehouse. Every system is slower in the later session, but not by a common factor: on the square, F-LOAM moves by 11% and KISS-ICP by 103%. Unlike the other figures in this section, this one pools every capture, including those the criteria of Section 3.5.5 exclude, because the quantity plotted is cost rather than accuracy. Each system is also drawn with its own marker, so that it can be identified without relying on colour.

That dependence on the session is itself a result. Between the two sessions, nine days apart, the trajectory error of every system reproduces to within 1.3 mm, the one exception being liorf at 4 mm, while the cost of the same work moves by anything between 4% and 103%. Nor does it move by a common factor: on the square, F-LOAM slows by 11% and KISS-ICP by 103%, so the penalty is a property of the implementation as much as of the board. The machine benchmark taken before each capture does not see any of it, moving by 2% between the two members of a pair. On this platform, accuracy is reproducible and cost is not, as Figure 9 shows.

The consequence is visible in the table. In the cooler session all six systems held the 100 ms budget at this site; in this one KISS-ICP does not, taking 105 ms per scan on the square in all three repeats and consuming 80% of its input rather than 99%. Whether a system meets its deadline on a passively cooled device is not a property of the system alone, but of the session it is run in.

### 4.4. Embedded—Warehouse

**Figure 10:**
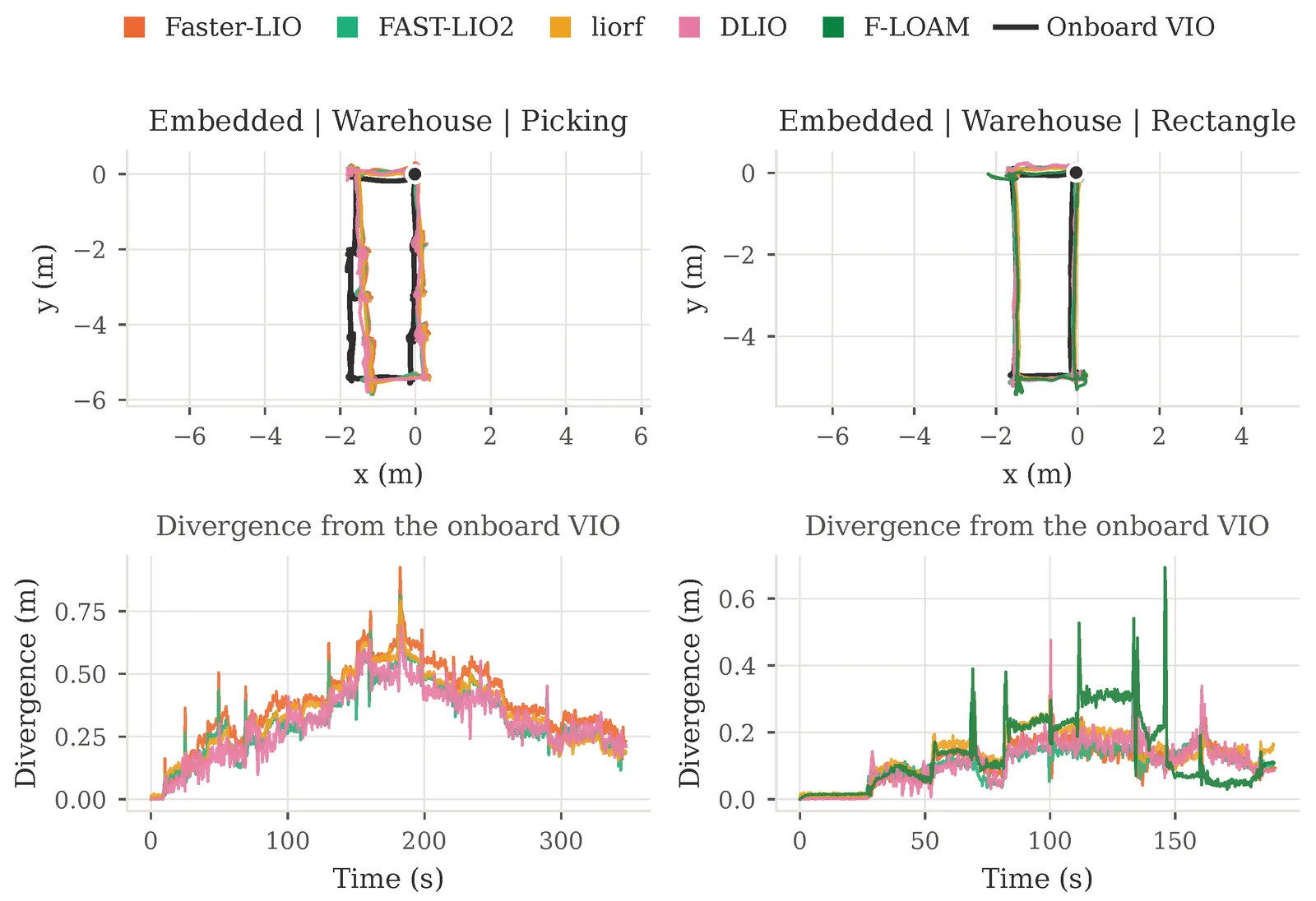
The same warehouse recordings on the embedded platform, against the same visual-inertial estimate. Only captures admitted by the criteria of Section 3.5.5 are drawn, so each system that fails is absent from the panel in which it failed: KISS-ICP, which discards between a half and two-thirds of its input at this site, appears in neither, and F-LOAM is absent from the picking trajectory, where it queues. What remains is the set of systems that continue to operate on the target, and they agree with one another there as they do on the workstation. The black marker indicates the start of each trajectory.

Replaying the warehouse recordings on the embedded platform, reported in Table 8, combines the two effects above, and it is where the ranking obtained on the workstation stops holding. Four systems continue to operate, as Figure 10 shows; two fail, by opposite mechanisms.

F-LOAM processes every scan it is given and falls progressively further behind, because its callbacks only append each scan to an unbounded queue that a separate thread drains. On the picking trajectory its end-to-end interval reaches 6.3 s, sixty-three times the interval between scans, and its resident memory grows to 1.66 GB; on the aisle traverse the same behaviour gives 2.0 s and 680 MB. Those figures are the depth of an unbounded queue rather than the cost of a scan, and the cost itself has to be recovered from the rate at which the system falls behind, as Section 3.5.3 describes. This failure is loud: the latency, the coverage and the memory all report it independently, and any one of them would be enough.

**Figure 11:**
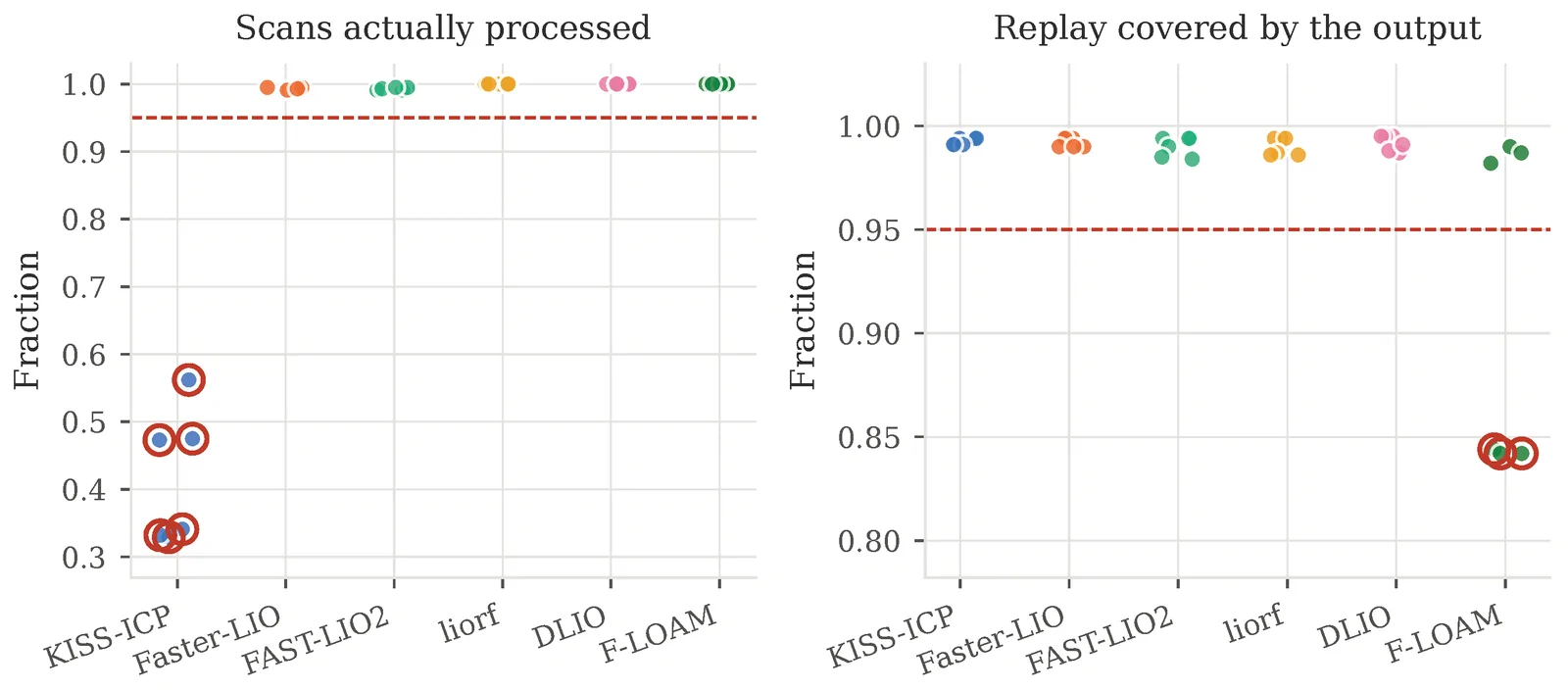
Every capture of the rectangle and picking recordings on the embedded platform against two of the admission criteria. The system that queues fails on coverage alone and the system that discards input fails on the fraction of scans processed alone, so neither criterion detects both. By coverage, the captures in which KISS-ICP processed a third to a half of its input are indistinguishable from working ones. Each point is one capture, coloured by system as in the other figures; the dashed red line marks the 95% admission threshold, and captures that fall below it are circled in red.

KISS-ICP does the opposite. Its ROS interface subscribes with a queue of a single message and registers each scan inside the callback, so once registration takes longer than the interval between scans, every scan that arrives in the meantime is replaced by the next without notice. It consumes 33% of the scans on the picking trajectory, 47% on the rectangle and 50% on the aisle. Every other indicator remains nominal: its latency does not grow, it spans essentially the whole of each recording, and its memory remains the lowest in the study, unchanged from the runs in which it was working correctly. On the aisle traverse the harness reports it as keeping up, and it is keeping up—it is current precisely because it threw half the scans away. Of the four criteria of Section 3.5.5, exactly one detects this, and Figure 11 is that criterion against the one that does not.

The estimate collapses with the input. KISS-ICP diverges from the reference by 0.945 m on the picking trajectory, against 0.146 m for the same system and configuration on the workstation, and by 2.739 m on the rectangle. The system that was the most accurate in the study at the laboratory is the one that diverges furthest from the reference at the held-out site on the target, and it became so without any indicator other than the fraction of consumed input changing.

The consequence for selection is direct. On the workstation the Pareto front held two systems, KISS-ICP and Faster-LIO. On the target, at a site neither was tuned on, one of those two fails, consuming a third to a half of its input. Two of the six, Faster-LIO and DLIO, are inexpensive, within budget and consistent with the reference at both sites and on both machines, and the evidence separates them in one direction. At the laboratory, the only site whose reference can rank them, Faster-LIO scores 0.037 and 0.040 m on the target against 0.047 and 0.046 m for DLIO, and replayed in one session on the board it takes 20 to 30 ms per scan against 30 to 39 ms and occupies 0.42 of a core against 0.54. DLIO leads on memory alone, on four of the five recordings.

### 4.5. Selected System vs. Onboard Estimator—Replay

The vehicle does not arrive without a localisation system. It already carries a visual-inertial estimator, and the question a practitioner faces is therefore not which LiDAR system is the best of six, but whether adding one is worth the processor it costs. That comparison is possible only at the laboratory, where motion capture and the visual-inertial estimate were recorded at the same time and both can be scored against the same truth.

Table 9 gives it for Faster-LIO, the system selected in Section 4.4. Typical error falls to roughly a third: from 0.103 m to 0.037 m on the square and from 0.102 m to 0.040 m on the picking trajectory. The worst-case error falls by considerably less, from 0.227 m to 0.167 m and from 0.237 m to 0.224 m, so the improvement is in the error the vehicle carries most of the time rather than in the excursions it must be designed to tolerate. Both figures should be read together: a system that reduces typical error to a third while leaving the tail largely intact changes what a mission can assume on average, not what it must be safe against.

Two asymmetries qualify the comparison, and neither can be removed. The visual-inertial estimate was produced live during the flight, at 30 Hz, on a computer that was simultaneously running the flight services and recording the data; Faster-LIO was replayed afterwards at the 10 Hz of the sensor, on the same computer but without the recording load. And the two use different sensors, so what is compared is not two algorithms but two localisation systems as they would be deployed. That is the comparison a deployment decision needs, and it is the reason it is reported here despite not being like for like.

### 4.6. Selected System vs. Onboard Estimator—Closed Loop

**Figure 12:**
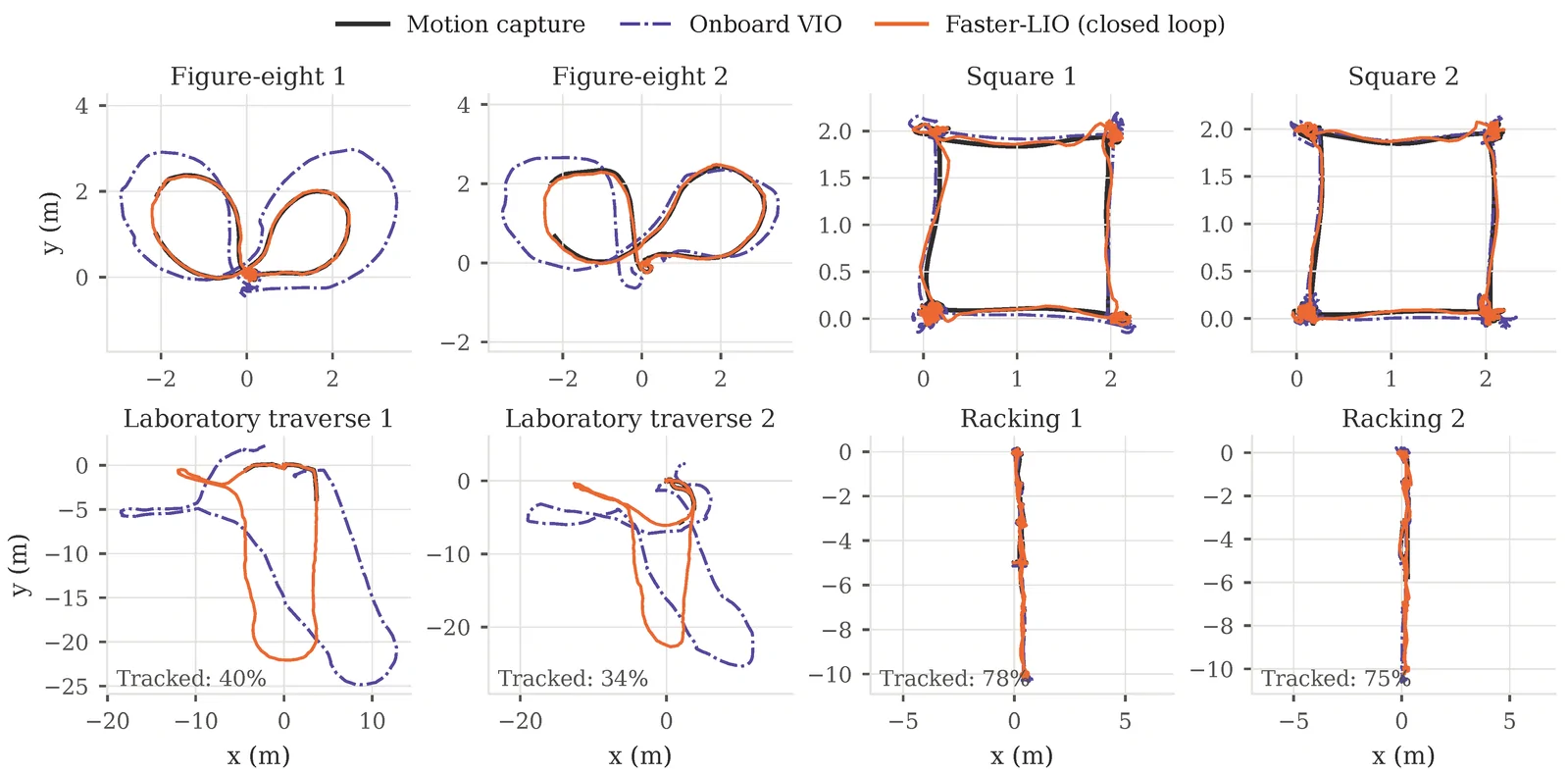
The eight closed-loop flights seen from above, with Faster-LIO in the control loop and the visual-inertial estimate recorded alongside it, each rigidly aligned to motion capture. On the racking and laboratory traverse flights the reference covers only the part flown inside the tracked volume, and the percentage shown in those panels is the fraction of the flight tracked by motion capture, over which the error is computed (Table 3).

The eight flights of Table 3 were flown with Faster-LIO running on the embedded platform and supplying the position fused by the autopilot, so that, unlike in replay, the estimate was computed from the live sensor and fed back to the controller. All eight were completed on that estimate. Table 10 reports each against motion capture and Figure 12 shows the trajectories. The error of Faster-LIO lies between 0.054 and 0.210 m, with a median of 0.078 m, and is below that of the visual-inertial estimator, recorded in the same flights outside the control loop, on every one of them. The figure-eight trajectories carry the largest error, and they are the shortest flights and those with the least hovering. As in replay, the gain is in typical error rather than in the tail, and on both squares and on one racking flight the maximum error of Faster-LIO exceeds that of the visual-inertial estimate.

The square is the one manoeuvre shared with the replayed recordings. There, Faster-LIO scores 0.081 and 0.108 m in flight against 0.037 m in replay, while the visual-inertial estimate scores 0.110 and 0.120 m, close to the 0.10 m characterised in Section 3.2. The flights differ from the replayed recording in duration and were flown on a different day, and this data does not separate the effect of closed-loop operation from those differences.

The estimate kept pace with the sensor on every flight, publishing at 10.0 Hz with one pose per scan and no gap in the sequence of stamps. On the seven flights whose recording clock allows the measurement, the median interval from the arrival of a scan to the arrival of its pose was between 14.9 and 18.3 ms, with a 95th percentile of 22.6 to 32.2 ms and a worst case of 45.5 ms. Replay on the same board took 24.0 and 25.2 ms per scan on the laboratory recordings (Table 7), so replay did not understate the time cost, although the two differ in recording and thermal state. The flight recordings carry no resource monitor, so processor and memory are not available. Errors exclude the poses within 1 s of any interruption of the reference, because the first samples after the motion capture re-acquires the vehicle are unreliable.

## 5. Discussion

### 5.1. Implications for Evaluating These Systems

The results above separate cleanly into quantities that survive a change of machine and quantities that do not. An accuracy ranking obtained on a workstation is likely to hold on an embedded target, provided the target is fast enough for the systems to consume their input. A cost ranking obtained on a workstation is not, and it fails in a known direction rather than randomly: it favours implementations that distribute their work across threads, which are the ones with the most to lose when the core count falls. A reader cannot correct for this by scaling, because the factor differs per system.

On a passively cooled device, a single cost figure describes one session rather than a system. Reporting one is therefore incomplete unless the session is identified, and neither of the two quantities that might correct a comparison across sessions does so. The fixed-work benchmark sampled before each capture moved by 2% while the cost moved by up to a factor of two, and the processor temperature, although recorded, does not account for the shift, since a later session ran Faster-LIO 5 to 12% faster at the same temperature. Costs are therefore compared across systems only within one session, which is why DLIO is set against Faster-LIO replayed alongside it rather than against the other rows.

Any evaluation that reports a rate (poses per second, real-time factor, latency per scan) should report alongside it the fraction of the input that produced it, since without that fraction a system that discards most of its input can appear to be the fastest.

### 5.2. Choosing a System, and What the Aisle Does Not Settle

Taken together, the measurements support one selection. Two of the six systems are inexpensive, within the sensor budget and consistent with the reference at both sites and on both machines, and of those two, Faster-LIO is the more accurate at the laboratory and the cheaper on the target in time and processor, with DLIO ahead on memory alone, on four of the five recordings. Faster-LIO reduces the typical error of the estimator the vehicle already carries to roughly a third at between 0.4 and 0.6 of a core, which is affordable on a computer with four cores available and with flight services occupying nearly two of them, and, flown as the vehicle’s position source, it kept pace with the sensor and stayed below the error of that estimator on all eight flights.

The aisle traverse, the recording closest to the deployment, carries no reference, and for it this study establishes what the systems cost and which of them keep up, not which of them is right. Rows of racking that repeat along the direction of travel leave the along-corridor direction weakly constrained [2,44], so accuracy measured at the laboratory, where racking runs along one side only, is not evidence of accuracy along such an aisle.

That limit is intrinsic to odometry rather than to any of the implementations. A system estimating incremental motion without closing loops interprets an environment of this kind as an infinite corridor [27], and only one of the six evaluated is capable of closing a loop at all. Whether the drift accumulated over a full aisle is tolerable for inventory work, and whether loop closure is required to bound it, are questions this data can pose, but not answer.

### 5.3. Limitations

The evaluation rests on two sites, one sensor, one vehicle and one embedded platform, and its conclusions are bounded accordingly. The parameters of every system were selected based on the laboratory recordings alone; the warehouse recordings were held out, but the reference available there carries error of the same order as the quantities scored against it, so that site establishes whether a system continues to operate rather than how accurately it does so. The aisle traverse carries no reference at all.

The systems were evaluated by replaying recorded data rather than in the vehicle’s control loop. This is what allows the two machines to be compared on identical input, and it is the protocol comparable benchmarks adopt, but it does not reproduce the interleaving of callbacks a live sensor would impose. The closed-loop flights of Section 4.6 cover that condition for the selected system alone, at the laboratory alone, and without a record of processor or memory. The flights are dominated by hovering, with sustained translation on only one of the five recordings, so no conclusion is offered about aggressive motion.

The sensor omits an azimuth sector of about 13°, centred within a degree of the vehicle’s direction of travel, from a fifth of its point clouds; that omission is identical for all six systems and bounds the accuracy attainable on this data without favouring any of them.

Finally, one observation remains characterised rather than explained. It is the variation of cost between sessions. The processor temperature was recorded and does not account for it, and the ambient conditions and the state of the cooling attachment were not recorded.

## 6. Conclusions

This work evaluated six open-source LiDAR odometry and SLAM systems on flights recorded in a motion-capture laboratory and in an operational warehouse, a setting for which, to the best of our knowledge, no public LiDAR odometry dataset with ground truth exists, and replayed identical data on a workstation and on the embedded computer the vehicle carries.

Accuracy transferred: every laboratory figure obtained on the embedded computer fell within 3 mm of its workstation counterpart for every system that consumed its input. Cost did not. Moving from twelve cores to four slowed the systems by factors of 1.8 to 3.9, according to how much of their work they distribute across threads, so a cost ranking obtained on a workstation reorders on the target. On the passively cooled target, cost also moved by up to a factor of two between sessions whose trajectories agreed within millimetres.

Under that ceiling, two systems exceeded the sensor budget by opposite mechanisms. F-LOAM processed every scan and queued without bound, which latency, coverage and memory all reveal. KISS-ICP, the most accurate system on the workstation, discarded up to two-thirds of its input, because its interface keeps only the most recent scan, while every conventional indicator read normally, and only the fraction of input consumed exposed the failure. Any benchmark that reports a rate should therefore also report the fraction of input that produced it.

Of the two systems that stayed inexpensive, within the sensor budget and consistent with the reference at both sites and on both machines, Faster-LIO was the more accurate at the laboratory and the cheaper on the target. It reduced the typical error of the onboard visual-inertial estimator to about a third, at 0.4 to 0.6 of one core, although its worst case improved by less. Flown as the vehicle’s position source, it kept pace with the sensor and stayed below that estimator’s error on all eight closed-loop flights.

Future work comprises flying the selected system in closed loop at the warehouse with its processor and memory recorded, and examining how the materials a warehouse presents, from wrapped pallets to bare and painted metal and specular surfaces, affect these systems. This study did not vary what the sensor sees, and operated it in single-return mode.

## Figures and Tables

**Table 1:** Design of the six evaluated systems, grouped by the modalities of Figure 1.

System	Modality	Feature Extraction	Estimator and Residual	Map (Structure and Query)	Loop Closure
F-LOAM [19]	LO	Edge and planar points	Nonlinear least squares, point-to-line and point-to-plane	Separate edge and planar clouds, *k*-d tree	No
KISS-ICP [20]	LO	None (voxel downsampling)	ICP, point-to-point, adaptive threshold	Voxel hash grid, voxel neighbourhood	No
FAST-LIO2 [21]	LIO	None (voxel downsampling)	Iterated error-state Kalman filter, point-to-plane	Incremental *k*-d tree (ikd-Tree)	No
Faster-LIO [22]	LIO	None (voxel downsampling)	Iterated error-state Kalman filter, point-to-plane	Incremental sparse voxels (iVox), voxel neighbourhood	No
liorf ^1^ [23,24]	LIO	None (removed from LIO-SAM)	Factor graph (iSAM2), point-to-plane	Sliding set of keyframe clouds, *k*-d tree	Yes
DLIO [25]	LIO	None (voxel downsampling)	Hierarchical geometric observer, plane-to-plane (GICP))	Keyframe submap, *k*-d tree	No

LO: LiDAR Odometry; LIO: LiDAR-Inertial Odometry; GICP: Generalized Iterative Closest Point. ^1^ liorf is the fork of LIO-SAM built and evaluated here. Section 3 describes how it differs from LIO-SAM.

**Table 2:** The five recordings, the reference available in each, and the role each plays in the evaluation. Extents are the bounding box of the reference trajectory.

Recording	Duration (s)	Extent (m)	Reference	Role
Laboratory, square	129	2.3×2.3×1.5	Motion capture	Accuracy, tuning
Laboratory, picking	212	0.7×7.5×3.1	Motion capture	Accuracy, tuning
Warehouse, rectangle	190	1.7×5.2×1.6	Visual-inertial	Held out
Warehouse, picking	347	1.9×5.6×4.6	Visual-inertial	Held out
Warehouse, aisle	322	1.6×∼40×4.0 ^1^	None	Held out, cost only

^1^ No reference exists for this recording; the extent is taken from the estimated trajectories.

**Table 3:** The four manoeuvres flown in closed loop, each repeated twice. Durations, extents and tracked fractions are given as the range over the two repetitions.

Manoeuvre	Duration (s)	Extent (m) ^1^	Tracked (%) ^2^	Path Flown
Square	184–211	2.3×2.2×1.7	100	A closed square of about 2 by 2 m, yawing to face the direction of travel, as in the laboratory square of Table 2
Figure-eight	85–89	5.6×2.8×1.4	98–99	Two lobes crossed at the centre, reversing the sense of yaw at each crossing, with the least hovering of the four
Racking	205–236	0.7×10.3×3.9	75–78	Lateral travel along about 10 m of shelving while facing it, over about 4 m of altitude
Laboratory traverse	164–201	16.3×23.1×4.3	34–40	A traverse of the laboratory rather than of the arena, the largest extent of the four

^1^ Bounding box of the estimated trajectory, given to indicate scale. ^2^ Fraction of the flight tracked by motion capture, over which the error is computed.

**Table 4:** The revision from which each system was built, the modifications applied to it, and the configuration at which it was evaluated.

System	Revision ^1^	Local Modifications	Range (m)	Tuned Parameters
F-LOAM	20062e3	Config, launch, odometry node	0.45–15.0	map_resolution 0.2 (swept, unchanged)
KISS-ICP	v0.3.0	Config only	0.45–15.0	voxel_size 0.4 (default 0.2)
FAST-LIO2	7cc4175	Config, launch, preprocessing, mapping, IMU handling	0.45–15.0	filter_size_map 0.2; filter_size_surf 1.2; max_iteration 3
Faster-LIO	ea0e091	Launch, mapping	0.45–15.0	filter_size_map 0.8; ivox_grid_resolution 1.2; filter_size_surf 1.2; max_iteration 3
liorf	1d777a6	Config, launch, projection, utilities	0.45–15.0	mappingSurfLeafSize 0.4 (default 0.2)
DLIO	fc8d183	Config, launch	^2^	voxelFilter resolution 0.4 (default 0.25)

^1^ Repositories, in the order of the table: https://github.com/wh200720041/floam (accessed on 27 August 2026), https://github.com/PRBonn/kiss_icp (accessed on 27 August 2026), https://github.com/hku-mars/FAST_LIO (accessed on 27 August 2026), https://github.com/gaoxiang12/faster-lio (accessed on 27 August 2026), https://github.com/YJZLuckyBoy/liorf (accessed on 27 August 2026), https://github.com/vectr-ucla/direct_lidar_inertial_odometry (accessed on 27 August 2026). KISS-ICP was built from the source archive of release v0.3.0 rather than from a repository checkout, and therefore carries a version rather than a revision. ^2^ DLIO exposes no maximum range parameter and could not be brought onto the common range limit.

**Table 5:** The six systems at the laboratory site on the workstation, over five repeats. Trajectory error is the RMSE after rigid alignment, taken as the median across repeats, with the full range given where it exceeds one millimetre. In each recording, bold marks the lowest trajectory error, time per scan, CPU and memory.

System	Absolute Trajectory Error (m)	Time per Scan (ms)	Scans Consumed	CPU (Cores)	Peak RSS (MB)
*Square*					
F-LOAM	0.0317	42.8	1.00	0.70	117
KISS-ICP	**0.0313**	14.2	1.00	1.54	**62**
FAST-LIO2	0.0413	**6.8**	1.00	0.48	200
Faster-LIO	0.0404 (0.0397–0.0408)	6.4	1.00	**0.38**	82
liorf	0.0394 (0.0384–0.0422)	13.2 ^1^	1.00	1.05	156
DLIO	0.0449	20.4	1.00	0.44	79
*Picking*					
F-LOAM	0.0658 (0.0658–0.0672)	39.4	1.00	0.66	109
KISS-ICP	**0.0354**	12.5	1.00	1.39	**63**
FAST-LIO2	0.0512 (0.0472–0.0512)	**6.0**	1.00	0.48	219
Faster-LIO	0.0404	6.4	1.00	**0.38**	85
liorf	0.0394	12.7 ^1^	1.00	1.05	160
DLIO	0.0429	19.5	1.00	0.44	79

^1^ Computation, with the two scans of structural input buffering removed; the end-to-end interval for this system is an order of magnitude larger. See Section 3.5.3.

**Table 6:** The six systems at the warehouse site on the workstation, over five repeats for the rectangle and picking trajectories and three for the aisle traverse. The quantity reported is divergence from the vehicle’s own visual-inertial estimate rather than error against a metric reference, taken as the RMSE after rigid alignment; the systems that agree to within the uncertainty of that estimate are not ordered by it. In each recording, bold marks the lowest time per scan, CPU and memory; divergence is not marked, for the same reason.

System	Divergence (m)	Time per Scan (ms)	Scans Consumed	CPU (Cores)	Peak RSS (MB)
*Rectangle*					
F-LOAM	0.133	57.5	1.00	0.88	138
KISS-ICP	0.116	17.1	1.00	2.16	**69**
FAST-LIO2	0.108	9.2	1.00	0.58	218
Faster-LIO	0.104	**7.7**	1.00	0.50	89
liorf	0.105	17.6 ^1^	1.00	1.26	159
DLIO	0.117	17.3	1.00	**0.44**	79
*Picking*					
F-LOAM	0.346	79.1	1.00	1.15	166
KISS-ICP	0.146	23.6	1.00	3.10	**84**
FAST-LIO2	0.143	8.9	1.00	0.56	245
Faster-LIO	0.149	**7.8**	1.00	0.60	99
liorf	0.141	18.4 ^1^	1.00	1.23	174
DLIO	0.149	20.8	1.00	**0.50**	**84**
*Aisle traverse*					
F-LOAM	^2^	71.4	1.00	1.07	161
KISS-ICP	^2^	17.2	1.00	2.34	**69**
FAST-LIO2	^2^	**5.5**	1.00	0.48	273
Faster-LIO	^2^	6.2	1.00	0.42	90
liorf	^2^	21.4 ^1^	1.00	1.29	179
DLIO	^2^	14.8	1.00	**0.39**	116

^1^ Computation, with the two scans of structural input buffering removed; the end-to-end interval for this system is an order of magnitude larger. See Section 3.5.3. ^2^ This recording carries no reference of any kind and contributes cost alone.

**Table 8:** The six systems at the warehouse site on the embedded platform, over three repeats, confined to the four large cores. Divergence is measured against the vehicle’s own visual-inertial estimate. Peak temperature is that of the large-core cluster, median across repeats. In each recording, bold marks the lowest divergence, time per scan, CPU and memory among the rows admitted as results, and, in the scans-consumed column, captures that consumed less than 95% of their input.

System	Divergence (m)	Time per Scan (ms)	Scans Consumed	CPU (Cores)	Peak RSS (MB)	Peak Temp. (°C)
*Rectangle*						
F-LOAM	0.134	123.4 ^3^	1.00	1.30 ^3^	121 ^3^	96.2 ^3^
KISS-ICP	2.739 ^2^	146.9 ^4^	**0.47**	2.64 ^4^	76 ^4^	96.2 ^4^
FAST-LIO2	0.108	30.4	1.00	**0.36**	212	76.3
Faster-LIO	**0.105**	**27.5**	1.00	0.44	76	75.5
liorf	0.108	42.6 ^5^	1.00	0.86	130	81.3
DLIO ^6^	0.119	34.9	1.00	0.58	**59**	78.2
*Picking*						
F-LOAM	0.347	120.8 ^4^	1.00	1.50 ^4^	1659 ^4^	97.8 ^4^
KISS-ICP	0.945 ^2^	257.7 ^4^	**0.33**	2.70 ^4^	75 ^4^	96.6 ^4^
FAST-LIO2	0.144	35.0	1.00	**0.40**	225	74.7
Faster-LIO	0.149	**31.7**	1.00	0.62	85	84.5
liorf	**0.140**	48.5 ^5^	1.00	0.96	130	95.4
DLIO ^6^	0.150	39.2	1.00	0.62	**63**	77.8
*Aisle traverse*						
F-LOAM	^1^	110.3 ^4^	1.00	1.48 ^4^	680 ^4^	97.0 ^4^
KISS-ICP	^1^	200.8 ^4^	**0.50**	2.68 ^4^	50 ^4^	96.6 ^4^
FAST-LIO2	^1^	25.1	1.00	**0.30**	239	77.0
Faster-LIO	^1^	**24.4**	1.00	0.42	**79**	71.6
liorf	^1^	53.9 ^5^	1.00	1.04	148	88.8
DLIO ^6^	^1^	29.8	1.00	0.54	102	75.5

^1^ This recording carries no reference of any kind and contributes cost alone. ^2^ The system discarded most of its input on these recordings; the figure is reported for completeness and is not an accuracy result. ^3^ One of the three captures met the acceptance criteria of Section 3.5.5; these figures are that capture alone. ^4^ No capture of this system on this recording met the acceptance criteria; the figures characterise the failure and are not results. For F-LOAM the time per scan is recovered from the rate at which the system falls behind, because its end-to-end interval is the depth of its queue rather than the cost of a scan. ^5^ Computation, with the two scans of structural input buffering removed; see Section 3.5.3. ^6^ Replayed in a later session together with Faster-LIO, so its cost does not share the thermal state of the other rows.

**Table 9:** Faster-LIO and the vehicle’s existing visual-inertial estimator, both measured against motion capture on the two laboratory recordings. Faster-LIO was replayed on the embedded platform; the visual-inertial estimate was produced by the same computer in flight. Both figures are the RMSE over the associated poses, after the same rigid alignment. Bold marks the lower error of the two estimators.

Recording	Faster-LIO, RMSE (m)	VIO, RMSE (m)	Faster-LIO, Max (m)	VIO, Max (m)
Square	**0.037**	0.103	**0.167**	0.227
Picking	**0.040**	0.102	**0.224**	0.237

**Table 10:** Faster-LIO flown as the vehicle’s position source, scored against motion capture, with the visual-inertial estimate recorded in the same flight. Errors are the RMSE after rigid alignment; latency is the interval from the arrival of a scan to the arrival of its pose.

Flight	Faster-LIO RMSE (m)	VIO RMSE (m)	Latency, Median (ms)	Latency, 95th pct. (ms)
Figure-eight 1	0.172	0.559	15.9	23.2
Figure-eight 2	0.210	0.539	14.9	22.6
Square 1	0.081	0.110	– ^1^	– ^1^
Square 2	0.108	0.120	16.4	26.0
Racking 1	0.058	0.108	18.3	32.2
Racking 2	0.063	0.216	17.7	28.6
Laboratory traverse 1	0.054	2.360	17.1	29.1
Laboratory traverse 2	0.075	2.047	17.3	26.7

^1^ The recording clock of this flight drifted from the sensor clock, so latency is not measurable; the estimate was published at 10 Hz without gaps.

## Data Availability

The flight data acquired on the laboratory site used in this study are available at https://github.com/A-M4t0s/lidar_odometry_benchmark_dataset (accessed on 1 September 2026).

## Abbreviations

The following abbreviations are used in this manuscript:

CPU	Central Processing Unit
EKF	Extended Kalman Filter
FoV	Field of View
GNSS	Global Navigation Satellite System
GPU	Graphics Processing Unit
ICP	Iterative Closest Point
IMU	Inertial Measurement Unit
LiDAR	Light Detection and Ranging
LIO	LiDAR-Inertial Odometry
LO	LiDAR Odometry
MAP	Maximum a Posteriori
RMSE	Root Mean Square Error
ROS	Robot Operating System
SLAM	Simultaneous Localisation and Mapping
UAV	Unmanned Aerial Vehicle
VIO	Visual-Inertial Odometry
